# Comparative study of Danshen and Siwu decoction based on the molecular structures of the components and predicted targets

**DOI:** 10.1186/s12906-021-03209-1

**Published:** 2021-01-22

**Authors:** Yang Li, Li Qiao, Cong Chen, Zhenguo Wang, Xianjun Fu

**Affiliations:** 1 College of Intelligence and Information Engineering, Shandong University of Traditional Chinese Medicine, Ji’nan, 250355 Shandong China; 2 Experimental Center, Shandong University of Traditional Chinese Medicine, Ji’nan, 250355 Shandong China; 3 Institute of Traditional Chinese Medicine Literature and Culture, Shandong University of Traditional Chinese Medicine, Ji’nan, 250355 Shandong China; 4 Center for Marine Traditional Chinese Medicine Research, Qingdao Academy of Chinese Medical Science, Qingdao, 260000 Shandong China; 5 Laboratory of Traditional Chinese Medicine Network Pharmacology, Shandong University of Traditional Chinese Medicine, Ji’nan, 250355 Shandong China; 6 Shandong Research Center of Engineering and Technology for omics of TCM, Ji’nan, 250355 Shandong China

## Abstract

**Background:**

The sentence of “Danshen (Salvia Miltiorrhizae Radix et Rhizoma) and Siwu decoction are similar in function” was first recorded in an ancient Chinese medical book “Fu Ren Ming Li Lun”. This theory has far-reaching influence on the clinical practice of Chinese medicine and is highly respected by Chinese medical doctors. However, the theory has limitations and controversial part for there is no in-depth and system comparative study.

**Methods:**

We collected the molecular structures of 129 compounds of Danshen and 81 compounds of Siwu decoction from the literatures. MACCS fingerprints and Tanimoto similarity were calculated based on the molecular structures for comparing the structural feature. Molecular descriptors which represent physical and chemical properties were calculated by Discovery Studio. Principal component analysis (PCA) of was performed based on the descriptors. The ADMET properties were predicted by FAF-Drugs4. The effect targets for the compounds with good ADMET properties were confirmed from experimental data and predicted using the algorithm comprising Bernoulli Naive Bayes profiling.

**Results:**

Based on the molecular structures, the presented study compared the structural feature, physical and chemical properties, ADMET properties, and effect targets of compounds of Danshen and Siwu decoction. It is found that Danshen and Siwu decoction do not have the same main active components. Moreover, the 2D structure of compounds from Danshen and Siwu decoction is not similar. Some of the compounds of Danshen and Siwu decoction are similar in 3D structure. The compounds with good ADMET properties of Danshen and Siwu decoction have same predicted targets, but some have different targets.

**Conclusions:**

It can be inferred from the result that Danshen and Siwu decoction have some similarities, but also present differences from each other in the structure of the compounds and predicted targets. This may be the material basis of the similar and different traditional efficacy of Danshen and Siwu decoction. The setence of “ Danshen and Siwu decoction are similar in function. “ which is used in clinical has its material basis and target connotation to some extent. However, the traditional effects of Danshen and Siwu decoction are not exactly the same.

**Supplementary Information:**

The online version contains supplementary material available at 10.1186/s12906-021-03209-1.

## Background

Danshen is commonly used in Chinese medicine. Danshen as a Chinese medicine is the dried rhizoma of the plant *Salvia miltiorrhiza Bge*. It was first recorded in “Shennong Ben Cao Jing” and is one of 40 large-scale medicinal materials. Danshen is a kind of medicine used for treating disease of the heart and liver, which is bitter and slightly cold in nature according to TCM theory. It has the effects of promoting blood circulation, relieving pain, clearing heart and eliminating trouble, cooling blood and eliminating phlegm [1]. Danshen is widely used in the clinical practice of traditional Chinese medicine. According to statistics, there are 1007 prescriptions containing Danshen in the “Dictionary of Traditional Chinese Medicine Formula” [2]. The “Chinese Pharmacopoeia (2015)” edition contains 127 kinds of Chinese patent medicines of Danshen, accounting for about 10% of the total [1].

Siwu decoction was originally recorded in the “Tai ping hui min he ji ju fang”, which was boiled from four kinds of herbs: Dihuang, Danggui, Baishao, and Chuanxiong (Table 1). These four traditional Chinese medicines which are recorded in the Chinese Pharmacopoeia are common traditional Chinese medicinal materials. After long-term clinical practice, Siwu decoction has been proven to be effective in nourishing the liver, regulating blood circulation and strengthening [3]. “Pu Fu Zhou Medical Experience” evaluates Siwu decoction as “a prescription for all blood diseases” [4].

**Table 1:** The information of Chinese medicines involved in the study

Chinese medicine name	English name	Species (Latin)	Plant part
Danshen	Salvia Miltiorrhizae Radix et Rhizoma	*Salvia miltiorrhiza Bge.*	rhizoma
Dihuang	Rehmanniae Radix	*Rehmannia glutinosa Libosch.*	root
Danghui	Angelicae Sinensis Radix	*Angelica sinensis (Oliv.) Diels*	root
Baishao	Paeoniae Radix Alba	*Paeonia lactiflora Pall.*	root
Chuangxiong	Chuanxiong Rhizoma	*Ligusticum chuanxiong Hort.*	rhizoma

In the theory of “Fu ren ming li lun”, “ Siwu decoction cures women’s diseases, regardless of the amount of menstrual water before and after childbirth, can be replaced by Danshen, for the same treatment” [5]. According to “Ben cao hui yan”, Danshen has the effect of Siwu decoction [6]. These have formed the theory that “Danshen and Siwu decoction are similar in function. “, which produced a profound impact on the clinical practice of Chinese medicine. However, the theory has limitations and controversial part for there is no in-depth and system comparative study on Danshen and Siwu decoction.

It is well known that one source of original research of traditional Chinese medicine is the study of modern pharmacodynamic material basis based on the traditional efficacy of traditional Chinese medicine [7]. The compounds with similar structures and chemical properties may have similar biological function and target. This theory has been widely recognized and applied in medicinal chemistry [8]. For example, neuraminidase which binds to sialic acid is one of the targets of influenza treatment drugs. Based on the structure of sialic acid, the sialic acid analogue Zanamivir was obtained, which was approved by the FDA in 2009 as a clinical treatment for influenza [9].

In this study, we collected the molecular structures of the chemical constituents of Danshen and Siwu decoction from the literature. Based on molecular descriptors, the molecular structures of Danshen and Siwu decoction are compared in terms of similarities, ADMET properties and effect targets. We also compare the molecular scaffolds of Danshen and Siwu decoction. Based on the structure of the material composition, we attempt to compare Danshen and Siwu decoction systematically, revealing the scientific connotation of the theory of “Danshen and Siwu decoction are similar in function. “, and provide a scientific basis for the clinical application.

## Methods

### Data

In this study, we collected the molecular structures of the chemical constituents of Danshen and Siwu decoction from the literatures [5, 10–65]. The molecular structure of 129 compounds of Danshen components and the molecular structure of 81 compounds of Siwu decoction components were confirmed (shown in Supplementary information). The structures of the main components of Danshen are tanshinone and derivatives, acid and derivatives and ester and derivatives. The main components in Siwu decoction are acids, esters, nitrogen-containing compound and polycyclic hydrocarbon, such as ferulic acid, ginger sugar ester, paeoniflorin, galloyl paeoniflorin [49]. The scaffolds and representative structures of Danshen and Siwu decoction are shown in Tables 2 and 3.

**Table 2:**
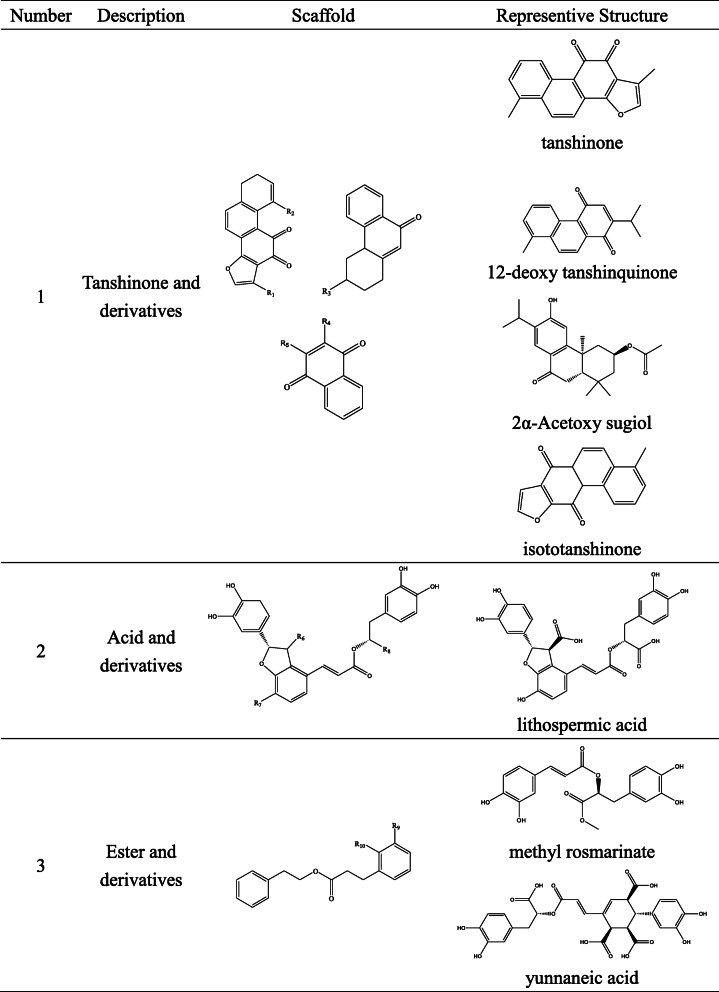
Scaffolds and representative structures of Danshen

**Table 3:**
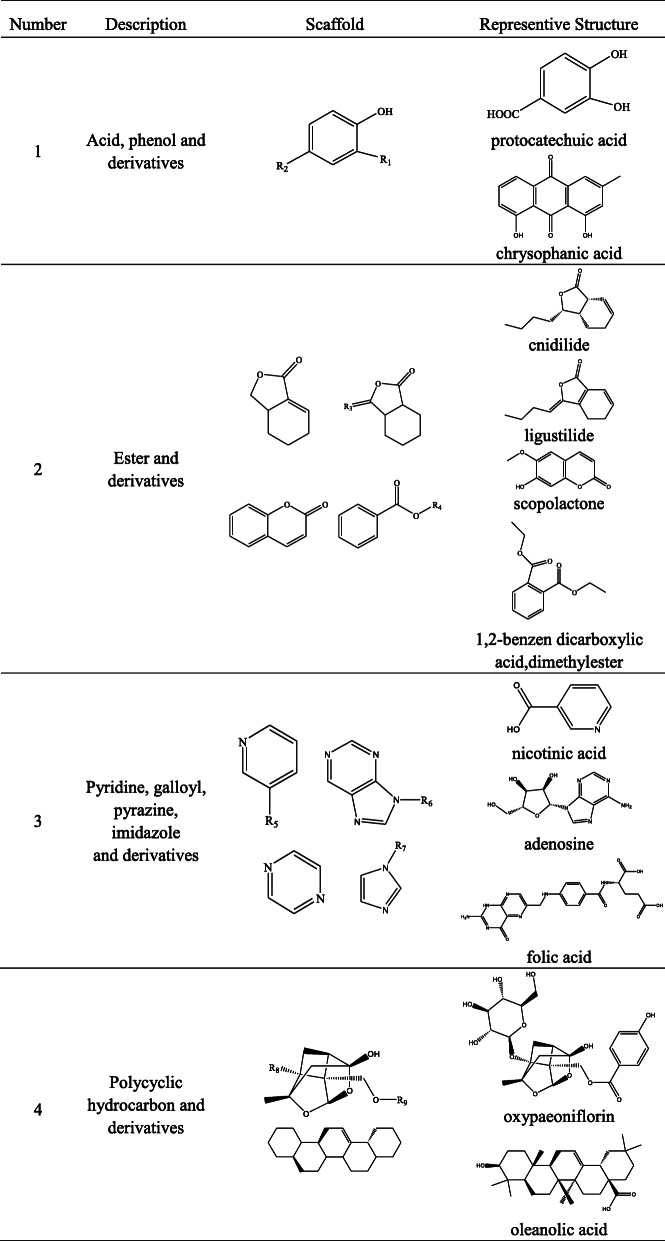
Scaffolds and representative structures of Siwu decoction

### Structural similarity comparison

For structural similarity comparison, based on the molecular structure of Danshen and Siwu decoction, MACCS fingerprints describing the 2D molecular structure were calculated.

The MACCS fingerprint is a descriptor that contains 166 codes representing the molecular structure “0” or “1”. The “0” indicates that the specified substructure does not exist in the compound, and the “1” indicates that the specified substructure exists. The Tanimoto similarity coefficient based on the MACCS fingerprint were calculated through through Eq. (1) to represent the similarity.

1 $$ \mathrm{T}\left(\mathrm{x},\mathrm{y}\right)=\frac{\sum {\boldsymbol{x}}_{\boldsymbol{i}}{\boldsymbol{y}}_{\boldsymbol{i}}}{\sqrt{\sum {\boldsymbol{x}}_{\boldsymbol{i}}^{\mathbf{2}}}+\sqrt{\sum {\boldsymbol{y}}_{\boldsymbol{i}}^{\mathbf{2}}}-\sum {\boldsymbol{x}}_{\boldsymbol{i}}{\boldsymbol{y}}_{\boldsymbol{i}}} $$

Where x_i_, y_i_ is two vectors generated by MACCS fingerprints, respectively. The differences between the 2D structures of Danshen and Siwu decoction can be compared by MACCS fingerprint.

### Comparison of physical and chemical properties

Molecular descriptors were calculated by Discovery Studio (2017R2), including 2D descriptors and 3D descriptors. 2D descriptors included number of atoms, molecular weight, ALogP and so on. 3D descriptors included Dipole, Jurs Descriptors, Principal Moments of Inertia, Shadow Indices and Propgen Properties [66]. Dipole properties have been associated with the recognition and binding of ligand and receptor. Jurs Descriptors combines the shape and electronic information of a molecule. These descriptors calculate the partial charge of an atom onto the solvent accessible surface area of individual atoms [67]. Principal Moments of Inertia characterizes the size of a molecule and calculates the inertia of the principal axes [68]. Shadow Indices is a type of topology descriptor representing the shape of a molecule [69]. The ADMET properties of Danshen and Siwu decoction were also predicted by FAF-Drugs4, including Molecular Weight (MW), logP, logD, logSw, topological Polar Surface Area (tPSA), Hydrogen Bond Donnors (HBD), Hydrogen Bond Acceptors (HBA), etc. [70].

### Target prediction

For the compounds with good ADMET properties, we look for the confirmed targets from experimental data. And for the compounds with good ADMET properties but no experimental data, calculations of predicted targets were performed. According to the chemical structure of the components, the algorithm comprising Bernoulli Naive Bayes profiling was used to predict the target of Danshen and Siwu decoction [71, 72]. The model covered 195 million bioactivity data from ChEMBL [73] and PubChem [74]. It predicted the target of each compound through full set of pathways from NCBI BioSystems [75].

## Results

### Structural similarity comparison

**Fig. 1:**
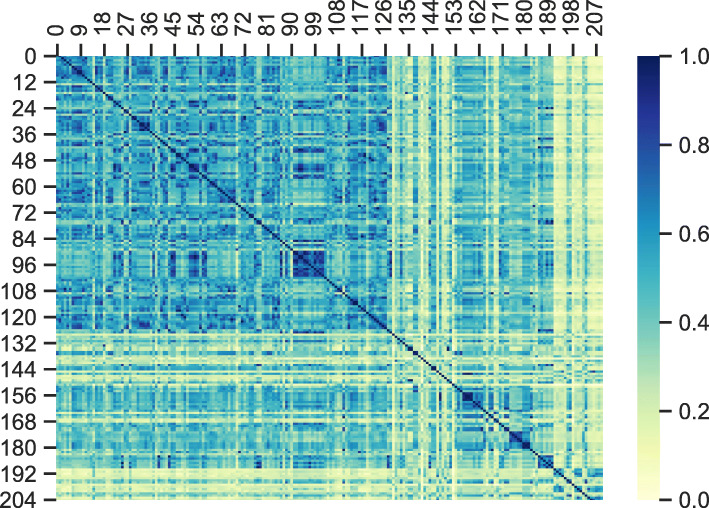
The hot map for similarity comparative result of Danshen and Siwu decoction, where No.1–129 represents the compound in Danshen, No.130–210 represents the compound in Siwu decoction

In the compound of Danshen and Siwu decoction, ferulic acid, caffeic acid and protocatechuic acid are all present. In order to calculate the similarity, MACCS fingerprints were used for the compounds. The similarity comparative results are shown in Fig. 1. The similarities of Danshen and Siwu decoction based on MACCS fingerprints are concentrated at 0.3–0.4. There are 1841 of 9916 (18.57%) of the similarities based on MACCS fingerprints more than 0.5. The number of 1261 (12.72%) of the similarities are in the range of [0.5, 0.6). The number of 480 (4.84%) of the similarities are in the range of [0.6, 0.7). The number of 74 (0.74%) of the similarities are in the range of [0.7, 0.8). The number of 18 (0.18%) of the similarities are in the range of [0.8, 0.9). The number of 8 (0.08%) of the similarities are in the range of [0.9, 1.0). The 2D structure of the compounds from Danshen and Siwu decoction is not similar based on the results of of MACCS fingerprints calculation.

### Comparison of physical and chemical properties

**Fig. 2:**
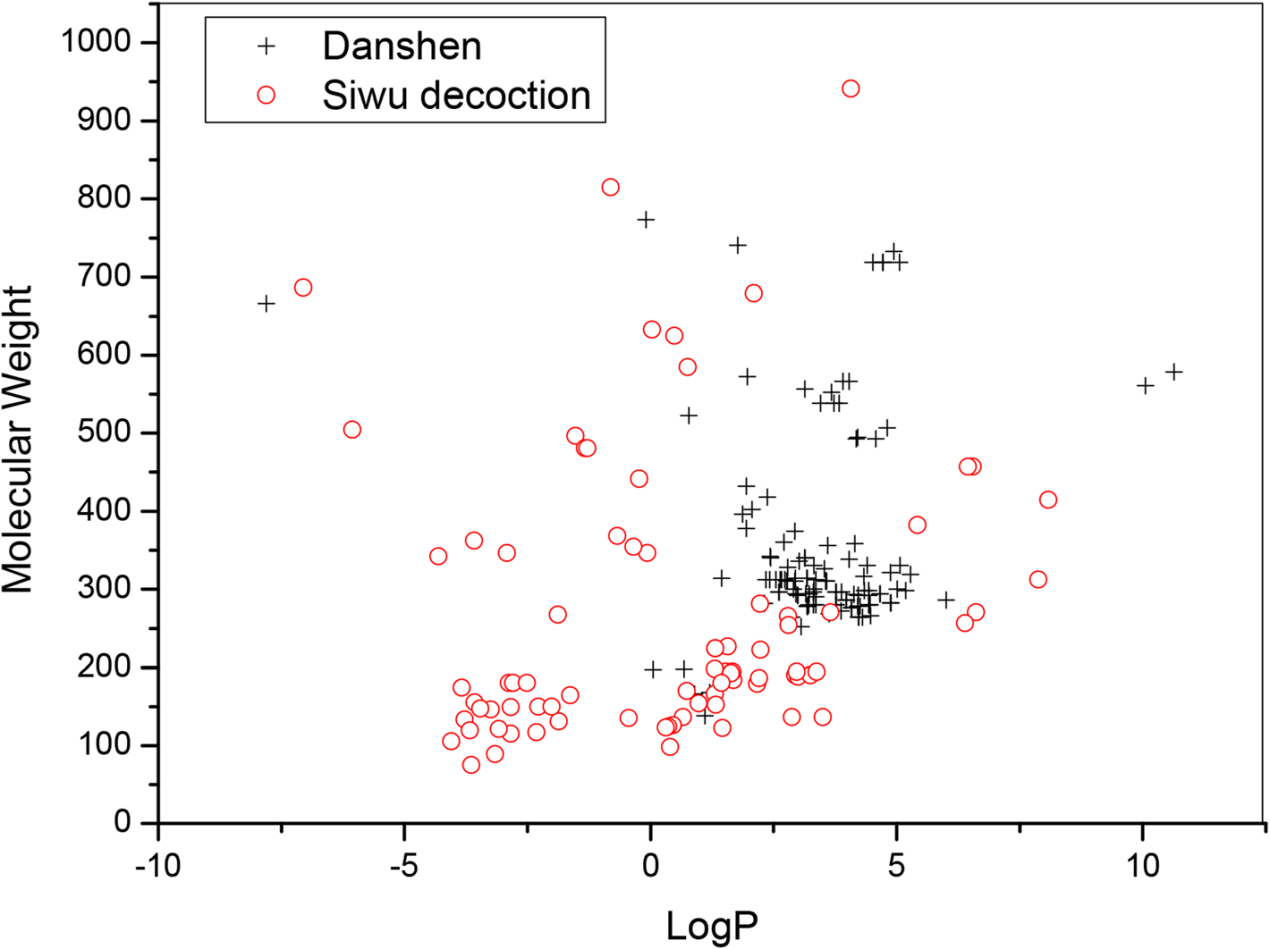
The scattered distributions of MW and LogP of Danshen and Siwu decoction

We calculated the basic physical and chemical properties based on the molecular structures by Discovery Studio [66]. The scattered distributions of MW and LogP of Danshen and Siwu decoction are shown in Fig. 2. For the compounds of Danshen, the molecular weight (MW) range is 138–774. For Siwu decoction, the compounds have a molecular weight (MW) range of 75–941. The range of LogP for Danshen is − 7.85-10.63. For Siwu decoction, the range of LogP is − 7.06-8.08. The compounds in Danshen and Siwu decoction are somewhat similar in basic physical and chemical properties.

**Fig. 3:**
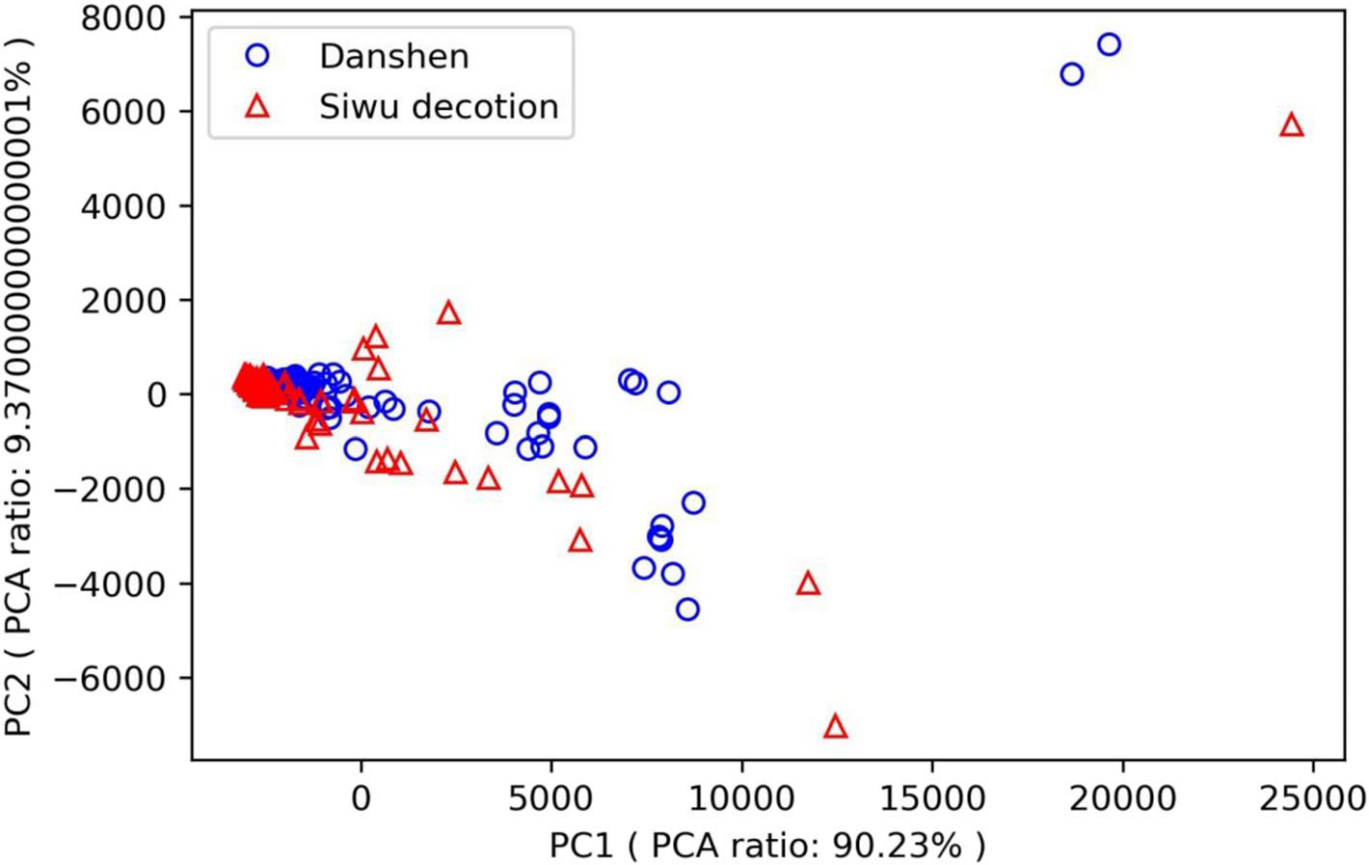
The chemical space characterized by the first two principal components obtained by PCA for compounds of Danshen and Siwu decoction

The method of principal component analysis (PCA) of based on the 3D descriptors was used to represent the chemical space of Danshen and Siwu decoction. The first two principal components obtained by PCA were used to describe the molecules. The chemical space characterized by the first two principal components from the method of PCA based on 3D descriptors for Danshen and Siwu decoction are shown in Fig. 3. From the PCA analysis, it can be found that the cumulative contributions of the two resulting principal eigenvectors (PC1 and PC2) are 90.23 and 9.37%, respectively. The accumulation of PC1 and PC2 reaches 99.60%, which indicates that results of PCA based on the 3D descriptors can be used to represent the higher-dimensional data. The chemical spaces by PCA based on the 3D decriptors for Danshen and Siwu decoction almost completely covered each other. This shows that the 3D molecular structures of Danshen and Siwu decoction compounds are somewhat similar.

### ADMET properties prediction

**Fig. 4:**
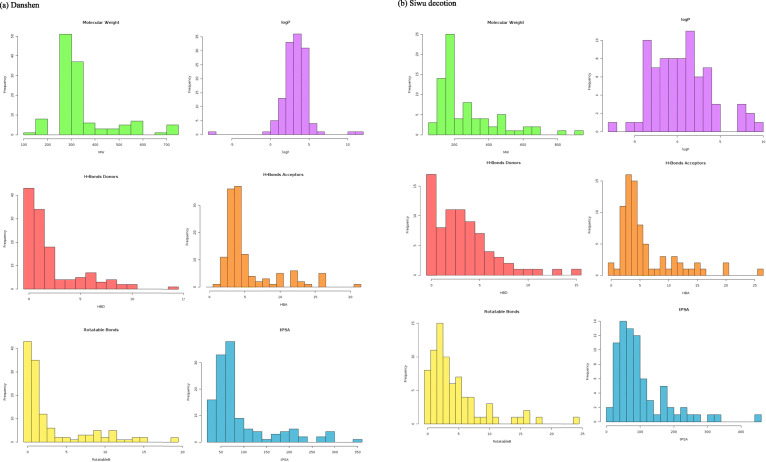
The distributions diagrams of the values of logP, tPSA, Molecular Weight, Rotatable Bonds, H-Bonds Acceptors and Donors computed for the main components of (a) Danshen and (b) Siwu decoction

The computational predictions of some ADMET properties (Adsorption, Distribution, Metabolism, Excretion and Toxicity) for the main components of Danshen and Siwu decoction were calculated by FAF-Drugs4 [70]. The distributions diagrams of the values of logP, tPSA, MW, Rotatable Bonds, HBD and HBA computed for the main components of (a) Danshen and (b) Siwu decoction were shown in Fig. 4. For Danshen, the molecular weight of most compounds is around 300, logP is mostly concentrated at 0–5, HBD concentrated at 0–3, HBA is mostly concentrated at 2–5, most compounds have less than 3 rotatable bonds, and TPSA distribution is concentrated in 50–100. For Siwu decoction, the molecular weight of most compounds is below 200, logP is mostly concentrated at − 4-4, HBD concentrated at 0–5, HBA is mostly concentrated at 2–6, most compounds have less than 6 rotatable bonds, and TPSA distribution is concentrated below 100. The ADMET properties of compounds in Danshen and Siwu decoction are somewhat similiar.

### Target analysis

For the compounds with good ADMET properties, we look for the confirmed targets from experimental data. The targets of caffeic acid, ferulic acid, isoferulic acid, rosmarinic acid, vanillic Acid, protocatechuic acid, gallic acid, diethyl phthalate and ligustilide can be found on ChEMBL [73]. We have verified according to the original data from scientific literatures and PubChem BioAssays [74]. The compounds and their proven targets are shown in Supplementary information Table S1. For the compounds with reported experimental data, the caffeic acid has 221 targets including arachidonate 5-lipoxygenase related to inflammation [76], which is also the result of predicted target. The vanillic Acid, ferulic acid and Isoferulic acid has 45, 240 and 16 targets, respectively. The predicted target is arachidonate 5-lipoxygenase. There is no relevant experimental data to confirm this predicted target. The rosmarinic acid has 73 targets including aldose reductase related to diabetes mellitus [77], which is also the result of predicted target. The above compounds are from Danshen. The protocatechuic acid has 72 targets including carbonic anhydrase 2 related to glaucoma, osteoporosis, epilepsy, tumors [78], which is also the result of predicted target. Gallic acid, Diethyl phthalate and ligustilide have 153, 17 and 153 targets, respectively. The predicted target is integrase, lysosomal alpha-glucosidase and ubiquitin carboxyl-terminal hydrolase 1. There is no relevant experimental data to confirm these predicted targets. The above compounds are from Siwu decoction.

**Fig. 5:**
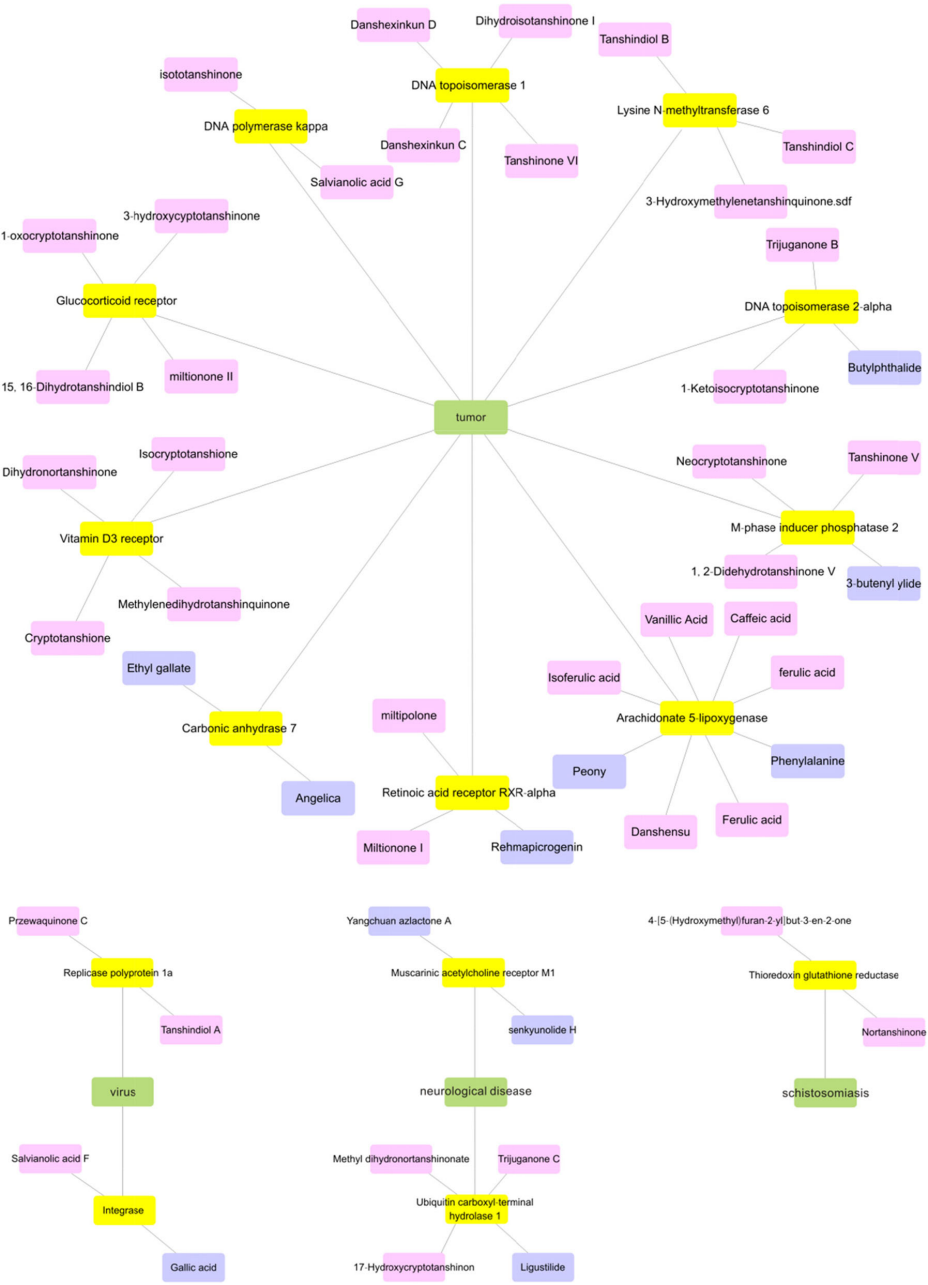
The predicted targets of partly compounds of Danshen and Siwu decoction. The compounds in Danshen are indicated in pink, the compound in Siwu decoction are indicated in blue, the predicted targets are indicated in yellow and the related disease are indicated in green

For the compounds with good ADMET properties but no experimental data, targets can be found by calculating predictions. The algorithm comprising Bernoulli Naive Bayes profiling [71, 72] was used to predict the target of the compounds. The most likely predicted target for each structure (greater than 0.9) was retained. We conducted a statistical analysis of the target of the compounds. The predicted targets of compounds with good ADMET predicted property of Danshen and Siwu decoction are shown in Fig. 5 and Table 4.

**Table 4:** The predicted targets of partly components of Danshen and Siwu decoction. Danshen

Target	Compoud	Disease	Source
Acetylcholinesterase	Tanshinaldehyde	Alzheimer’s disease	Danshen
Aldose reductase	Rosmarinic acid	diabetes mellitus	Danshen
Bcl-2-related protein EAT/mcl1	Neotanshinone A	leucocythemia	Danshen
Bile acid receptor	przewalskin A	cardiovascular disease, liver disease	Danshen
Carbonic anhydrase 2	Protocatechuic acid	tumour	Danshen
Cocaine esterase	Tanshinone II A Sodium Sulfonate	detoxification of xenobiotics and in the activation of ester and amide prodrugs	Danshen
Carbonic anhydrase	3-lactamide	tumor	Danshen
DNA topoisomerase 2	Trijuganone A	tumor	Danshen
M18 aspartyl aminopeptidase	Prolithospermic acid	parasitic disease	Danshen
Sex hormone-binding globulin	przewalskin B	diabetes mellitus	Danshen
TrpV4	3-Oxoisotaxodione	cardiovascular disease	Danshen
Adenosine receptor A1	Adenosine	central nervous system disease	Siwu decoction
Alpha-glucosidase, Glucoamylase	paeonin	hyperglycemia	Siwu decoction
Carbonic anhydrase 9	East terpene lactone	tumor	Siwu decoction
Cathepsin B	Leucine	tumor	Siwu decoction
Epidermal growth factor receptor	Vanillin	tumor	Siwu decoction
Integrin beta-1	Tyrosine	tumor	Siwu decoction
M-phase inducer phosphatase 1	Ligustilide A	tumor	Siwu decoction
Muscarinic acetylcholine receptor M2	Snake bed lactone	central nervous system disease	Siwu decoction
Sodium/glucose cotransporter 1	Rehmapicroside	diabetes mellitus	Siwu decoction
Transcriptional activator protein LasR	Senkyunolide J	tumor	Siwu decoction
Transient receptor potential cation channel subfamily A member 1	Dimethyl phthalate	neuroglioma	Siwu decoction

In Fig. 5, for the compounds without reported experimental data, multiple Danshen components act on targets DNA polymerase kappa, DNA topoisomerase 1, Glucocorticoid receptor, Lysine N-methyltransferase 6, Replicase polyprotein 1a, thioredoxin glutathione reductase, and Vitamin D3 receptor. Multiple Siwu decoction components act on targets Carbonic anhydrase 7 and arinic acetylcholine receptor M1. Among the above targets, DNA polymerase kappa, DNA topoisomerase 1, Carbonic anhydrase 7, Glucocorticoid receptor, Lysine N-methyltransferase 6 and Vitamin D3 receptor are related to tumor, arinic acetylcholine receptor M1 is related to neurological diseases, Replicase polyprotein 1a is relataed to virus and thioredoxin glutathione reductase is an essential parasite enzyme [79–86]. For the predicted target, Bile acid receptor and TrpV4 are the targets for cardiovascular disease. The result is consistent with the clinical effects of Danshen [87]. The predicted target types of compounds of Danshen and Siwu decoction are overlapped. They are predicted to act on some anti-tumor targets.

The predicted targets of multiple Danshen and Siwu decoction are Arachidonate 5-lipoxygenase, DNA topoisomerase 2-alpha, integrase, M-phase inducer phosphatase 2, retinoic acid receptor RXR-alpha, and Ubiquitin carboxyl-terminal hydrolase 1(UCH-L1). For the above predicted targets, Arachidonate 5-lipoxygenase, DNA topoisomerase 2-alpha, M-phase inducer phosphatase 2 and retinoic acid receptor RXR-alpha are related to tumor, integrase is an important target for virus, is related to skin diseases, and UCH-L1 is related to neurological diseases [88–93]. These are same predictive targets for compounds of Danshen and Siwu decoction.

## Discussion

In this study, we compared the structural feature, physical and chemical properties, ADMET properties, and effect targets of compounds of Danshen and Siwu decoction.

MACCS fingerprints were calculated based on the molecular structure of Danshen and Siwu decoction for structural similarity comparison. The similarity is concentrated at 0.3–0.4. The 2D structure of Danshen and Siwu decoction is not similar based on the results of MACCS fingerprints calculation. The basic physical and chemical properties were calculated based on the molecular structures. Principal component analysis (PCA) of the 3D descriptors was performed to identify the chemical space of Danshen and Siwu decoction. The chemical spaces for Danshen and Siwu decoction nearly completely covered each other. The result shows that the 3D molecular structures of Danshen and Siwu decoction compounds are somewhat similar. The 2D structure of Danshen and Siwu decoction is not similar, but the compounds are somewhat similar in basic physical and chemical properties and 3D structure.

The ADMET properites of the compoounds were calculated FAF-Drugs4 [70]. The molecules with good ADMET properties can be considered to meet the following conditions:

(i) Oral absorption estimation: the range of logP is − 2 to 5, the range of MW is 150 to 500, the range of tPSA is 20 to 150, the range of Rotatable Bonds is 0 to 10, the range of H-Bonds Acceptors is 0 to 10 and the range of Donors is 0 to 5 (http://fafdrugs4.mti.univ-paris-diderot.fr/index.html);
(ii) Oral bioiavailability evaluation: Lipinski’s RO5 [94]; Veber rules [95]; Egan rules [96];
(iii) Solubility: Solubility Forecast Index [97];
(iv) Drug safety: GSK 4/400 rule [98]; no structural alerts [99];

The targets of the compounds with good predictive properties of ADMET were focused. We look for the confirmed targets from experimental data. The targets of caffeic acid, ferulic acid, isoferulic acid, rosmarinic acid, vanillic Acid, protocatechuic acid, gallic acid, diethyl phthalate and ligustilide are comfirmed. Some confirmed targets are the same as predicted targets for these compounds. And for the compounds with good ADMET properties but no experimental data, the algorithm comprising Bernoulli Naive Bayes profiling [71, 72] was used to predict the target of the compounds. The most likely predicted target for each structure (greater than 0.9) was retained. The predicted target types of compounds of Danshen and Siwu decoction are overlapped. They are predicted to act on some anti-tumor targets, such as DNA polymerase kappa, DNA topoisomerase 1, Carbonic anhydrase 7, Glucocorticoid receptor, Lysine N-methyltransferase 6 and Vitamin D3 receptor. There are same predictive targets for compounds of Danshen and Siwu decoction, such as Arachidonate 5-lipoxygenase, DNA topoisomerase 2-alpha, integrase, M-phase inducer phosphatase 2, retinoic acid receptor RXR-alpha, and Ubiquitin carboxyl-terminal hydrolase 1(UCH-L1). The effect targets for Danshen and Siwu decoction have similarities.

## Conclusion

Based on molecular structures, the study aimed to scientifically compare Danshen and Siwu decoction which are similar in function but not the same. Danshen and Siwu decoction were compared systematically based on the molecular structure of the components, including the structural feature, physical and chemical properties, ADMET properties, and effect targets. The molecular structure of 129 compounds of Danshen components and the molecular structure of 81 Siwu decoction compounds were confirmed in the study. The 2D structure of Danshen and Siwu decoction is not similar, but parts of the compounds are similar in basic physical and chemical properties and 3D structure. We calculated the ADMET properties, and obtained several compounds have good oral absorption, oral bioiavailability, solubility and safety. The components of Danshen and Siwu decoction similar in structure, but there are also differences. These compounds were used for further target research. In the target research, it was found that that the compounds of Danshen and Siwu decoction have same effect targets, such as tumor-related targets, neurological-related targets, but they also have different targets. Through the studies and conclusions, it can be found that the theory of “Danshen and Siwu decoction are similar in function. “ which is used in clinical has scientific basis and connotation to some extent. However, the effects of Danshen and Siwu decoction are not exactly the same. This is significant for the use of traditional Chinese medicine on clinic.

## Supplementary Information

**Additional file 1: Table S1.** The compounds and the proven targets.

**Additional file 2:** The molecular structure of 129 compounds of Danshen components used in this study.

**Additional file 3:** The molecular structure of 81 compounds of Siwu decoction components.

## Data Availability

The molecular structures of compounds used in this study can be found in txt file named “Salvia miltiorrhiza.txt” and “Siwu detcotion.txt”. The compounds and their proven targets can be found in Table S1. The datasets used and/or analyzed for this study are available from the corresponding author by reasonable request.

## Abbreviations

MW: Molecular Weight
tPSA: Topological Polar Surface Area
HBD: Hydrogen Bond Donnors
HBA: Hydrogen Bond Acceptors
PCA: Principal component analysis
UCH-L1: Ubiquitin carboxyl-terminal hydrolase 1
