# Supplementary material for: Comparative study of Danshen and Siwu decoction based on the molecular structures of the components and predicted targets
Source: BMC Complement Med Ther. 2021 Jan 22;21:42. doi: 10.1186/s12906-021-03209-1 (PMC7821527; doi:10.1186/s12906-021-03209-1)
Supplement: Supplementary file 1 — Additional file 1: Table S1. The compounds and the proven targets. [file 12906_2021_3209_MOESM1_ESM.docx]

Table S1 The compounds and the proven targets.

| compounds | ChEMBL ID | Name |
| --- | --- | --- |
| caffeic acid | CHEMBL1293235 | Prelamin-A/C |
|  | CHEMBL5365 | DNA polymerase kappa |
|  | CHEMBL2366839 | Neofusicoccum luteum |
|  | CHEMBL5929 | Alanine aminotransferase 1 |
|  | CHEMBL4789 | Carbonic anhydrase VA |
|  | CHEMBL612788 | Lasiodiplodia theobromae |
|  | CHEMBL261 | Carbonic anhydrase I |
|  | CHEMBL612744 | Feline coronavirus |
|  | CHEMBL4159 | Endoplasmic reticulum-associated amyloid beta-peptide-binding protein |
|  | CHEMBL2367030 | Botryosphaeria dothidea |
|  | CHEMBL2146316 | Putative uncharacterized protein |
|  | CHEMBL3317 | 5-lipoxygenase |
|  | CHEMBL1293298 | Peripheral myelin protein 22 |
|  | CHEMBL3318 | Tyrosinase |
|  | CHEMBL3885594 | Importin subunit beta-1/Snurportin-1 |
|  | CHEMBL4586 | Seed lipoxygenase-1 |
|  | CHEMBL2366838 | Diplodia mutila |
|  | CHEMBL4078 | Acetylcholinesterase |
|  | CHEMBL2392 | DNA polymerase beta |
|  | CHEMBL1293234 | Putative fructose-1 |
|  | CHEMBL2093861 | Menin/Histone-lysine N-methyltransferase MLL |
|  | CHEMBL1687677 | Pancreatic triacylglycerol lipase |
|  | CHEMBL1293285 | Endonuclease 4 |
|  | CHEMBL1075257 | Large T antigen |
|  | CHEMBL2885 | Carbonic anhydrase III |
|  | CHEMBL6110 | Thioredoxin glutathione reductase |
|  | CHEMBL2186 | Carbonic anhydrase XIII |
|  | CHEMBL612602 | Ralstonia solanacearum |
|  | CHEMBL2367189 | Togninia minima |
|  | CHEMBL3729 | Carbonic anhydrase IV |
|  | CHEMBL4357 | Alpha-glucosidase MAL62 |
|  | CHEMBL5686 | 6-phospho-1-fructokinase |
|  | CHEMBL2094253 | Cyclooxygenase |
|  | CHEMBL4528 | Hyaluronidase-1 |
|  | CHEMBL2193 | Cerebroside-sulfatase |
|  | CHEMBL1641360 | BiP isoform A |
|  | CHEMBL614051 | OVCAR-4 |
|  | CHEMBL2367106 | Neofusicoccum parvum |
|  | CHEMBL612500 | Pseudomonas fluorescens |
|  | CHEMBL5763 | Cholinesterase |
|  | CHEMBL2487 | Beta amyloid A4 protein |
|  | CHEMBL614317 | M14 |
|  | CHEMBL215 | Arachidonate 5-lipoxygenase |
|  | CHEMBL613860 | AGS |
|  | CHEMBL1075138 | Tyrosyl-DNA phosphodiesterase 1 |
|  | CHEMBL399 | HeLa |
|  | CHEMBL614919 | SK-MEL-28 |
|  | CHEMBL612679 | Respiratory syncytial virus |
|  | CHEMBL392 | A549 |
|  | CHEMBL366 | Candida albicans |
|  | CHEMBL2146302 | Glutaminase kidney isoform |
|  | CHEMBL613864 | Gibberella zeae |
|  | CHEMBL312 | Arachidonate 5-lipoxygenase |
|  | CHEMBL614888 | RXF 631 |
|  | CHEMBL614067 | CAKI-1 |
|  | CHEMBL613508 | DU-145 |
|  | CHEMBL384 | HT-29 |
|  | CHEMBL378 | Human immunodeficiency virus 1 |
|  | CHEMBL2219 | Protein-tyrosine phosphatase LC-PTP |
|  | CHEMBL613102 | 786-0 |
|  | CHEMBL3396943 | DNA-3-methyladenine glycosylase |
|  | CHEMBL614803 | NCI-H322M |
|  | CHEMBL3513 | Acidic alpha-glucosidase |
|  | CHEMBL612877 | Leishmania amazonensis |
|  | CHEMBL614316 | HT-22 |
|  | CHEMBL3879801 | NON-PROTEIN TARGET |
|  | CHEMBL6035 | Thioredoxin reductase 1 |
|  | CHEMBL382 | CCRF-CEM |
|  | CHEMBL5896 | Lysine-specific demethylase 4A |
|  | CHEMBL397 | Jurkat |
|  | CHEMBL2367034 | Human herpesvirus 1 strain KOS |
|  | CHEMBL390 | PC-3 |
|  | CHEMBL3864 | Protein-tyrosine phosphatase 2C |
|  | CHEMBL4999 | Aldo-keto reductase family 1 member C4 |
|  | CHEMBL203 | Epidermal growth factor receptor erbB1 |
|  | CHEMBL3251 | Nuclear factor NF-kappa-B p105 subunit |
|  | CHEMBL613453 | Meloidogyne incognita |
|  | CHEMBL204 | Thrombin |
|  | CHEMBL613126 | Human parainfluenza virus 3 |
|  | CHEMBL352 | Staphylococcus aureus |
|  | CHEMBL612555 | OVCAR-8 |
|  | CHEMBL1947 | Thyroid hormone receptor beta-1 |
|  | CHEMBL614164 | SNB-19 |
|  | CHEMBL614516 | A498 |
|  | CHEMBL614214 | OVCAR-5 |
|  | CHEMBL1900 | Aldose reductase |
|  | CHEMBL3969 | Carbonic anhydrase VB |
|  | CHEMBL1293226 | Lysine-specific demethylase 4D-like |
|  | CHEMBL4681 | Aldo-keto-reductase family 1 member C3 |
|  | CHEMBL614860 | SF-539 |
|  | CHEMBL613984 | IGROV-1 |
|  | CHEMBL391 | Vero |
|  | CHEMBL230 | Cyclooxygenase-2 |
|  | CHEMBL6032 | Histone-lysine N-methyltransferase |
|  | CHEMBL3471 | Human immunodeficiency virus type 1 integrase |
|  | CHEMBL613855 | HCC 2998 |
|  | CHEMBL614809 | HOP-18 |
|  | CHEMBL612543 | SNB-78 |
|  | CHEMBL614637 | KETR3 |
|  | CHEMBL333 | Matrix metalloproteinase-2 |
|  | CHEMBL614019 | M19-MEL |
|  | CHEMBL3025 | Carbonic anhydrase VI |
|  | CHEMBL612320 | Human coxsackievirus B4 |
|  | CHEMBL1741220 | Bromodomain adjacent to zinc finger domain protein 2B |
|  | CHEMBL612796 | UACC-257 |
|  | CHEMBL614096 | LOX IMVI |
|  | CHEMBL3559673 | 2-heptyl-4(1H)-quinolone synthase PqsD |
|  | CHEMBL1973 | Tyrosinase |
|  | CHEMBL614642 | DMS-114 |
|  | CHEMBL389 | P388 |
|  | CHEMBL394 | HCT-116 |
|  | CHEMBL612546 | Molecular identity unknown |
|  | CHEMBL340 | Cytochrome P450 3A4 |
|  | CHEMBL613740 | Influenza A virus |
|  | CHEMBL614955 | Aspergillus flavus |
|  | CHEMBL614733 | NCI-H292 |
|  | CHEMBL612811 | WISH |
|  | CHEMBL614882 | RPMI-8226 |
|  | CHEMBL613606 | Aorta |
|  | CHEMBL335 | Protein-tyrosine phosphatase 1B |
|  | CHEMBL321 | Matrix metalloproteinase 9 |
|  | CHEMBL614387 | NCI-H522 |
|  | CHEMBL1293248 | 4'-phosphopantetheinyl transferase ffp |
|  | CHEMBL387 | MCF7 |
|  | CHEMBL614034 | DMS-273 |
|  | CHEMBL4040 | MAP kinase ERK2 |
|  | CHEMBL2095165 | Heat shock protein HSP90 |
|  | CHEMBL613889 | HOP-92 |
|  | CHEMBL1929 | Xanthine dehydrogenase |
|  | CHEMBL612656 | B16-F10 |
|  | CHEMBL614184 | MT2 |
|  | CHEMBL353 | Staphylococcus epidermidis |
|  | CHEMBL615022 | U-251 |
|  | CHEMBL614886 | RXF 393 |
|  | CHEMBL240 | HERG |
|  | CHEMBL3510 | Carbonic anhydrase XIV |
|  | CHEMBL614213 | OVCAR-3 |
|  | CHEMBL614068 | MDCK |
|  | CHEMBL612545 | Unchecked |
|  | CHEMBL359 | Bacillus subtilis |
|  | CHEMBL613741 | Punta Toro virus |
|  | CHEMBL396 | NCI-H460 |
|  | CHEMBL614285 | DLD-1 |
|  | CHEMBL614021 | Malme-3M |
|  | CHEMBL614177 | MOLT-4 |
|  | CHEMBL614561 | COLO 205 |
|  | CHEMBL614054 | SN12C |
|  | CHEMBL2326 | Carbonic anhydrase VII |
|  | CHEMBL3194 | Transthyretin |
|  | CHEMBL614922 | SK-MEL-5 |
|  | CHEMBL612518 | LNCaP |
|  | CHEMBL2362975 | No relevant target |
|  | CHEMBL614610 | UACC-62 |
|  | CHEMBL612262 | SW-620 |
|  | CHEMBL3594 | Carbonic anhydrase IX |
|  | CHEMBL206 | Estrogen receptor alpha |
|  | CHEMBL332 | Matrix metalloproteinase-1 |
|  | CHEMBL613712 | Radical scavenging activity |
|  | CHEMBL3114 | Sucrase-isomaltase |
|  | CHEMBL2635 | Dual specificity protein phosphatase 3 |
|  | CHEMBL3242 | Carbonic anhydrase XII |
|  | CHEMBL614632 | RBL-2H3 |
|  | CHEMBL5905 | Aldo-keto reductase family 1 member C1 |
|  | CHEMBL2366750 | Diplodia seriata |
|  | CHEMBL2608 | Lysosomal alpha-glucosidase |
|  | CHEMBL1075183 | Potassium channel subfamily K member 2 |
|  | CHEMBL613362 | Hepatocyte |
|  | CHEMBL3514 | LDL-associated phospholipase A2 |
|  | CHEMBL376 | Rattus norvegicus |
|  | CHEMBL2093865 | Histone deacetylase |
|  | CHEMBL1697668 | Solute carrier organic anion transporter family member 1B1 |
|  | CHEMBL613493 | Vaccinia virus |
|  | CHEMBL5619 | DNA-(apurinic or apyrimidinic site) lyase |
|  | CHEMBL612558 | ADMET |
|  | CHEMBL5847 | Aldo-keto reductase family 1 member C2 |
|  | CHEMBL614709 | KM12 |
|  | CHEMBL614388 | UO-31 |
|  | CHEMBL614361 | T47D |
|  | CHEMBL614997 | NCI-H23 |
|  | CHEMBL358 | Aspergillus niger |
|  | CHEMBL383 | HL-60 |
|  | CHEMBL1293224 | Microtubule-associated protein tau |
|  | CHEMBL3785 | Hydroxycarboxylic acid receptor 2 |
|  | CHEMBL4332 | Quinone oxidoreductase |
|  | CHEMBL1743121 | Solute carrier organic anion transporter family member 1B3 |
|  | CHEMBL205 | Carbonic anhydrase II |
|  | CHEMBL614300 | SR |
|  | CHEMBL356 | Streptococcus pyogenes |
|  | CHEMBL364 | Plasmodium falciparum |
|  | CHEMBL613725 | Sindbis virus |
|  | CHEMBL1293255 | 15-hydroxyprostaglandin dehydrogenase [NAD+] |
|  | CHEMBL614519 | ACHN |
|  | CHEMBL385 | K562 |
|  | CHEMBL612557 | RAW264.7 |
|  | CHEMBL612794 | U-937 |
|  | CHEMBL220 | Acetylcholinesterase |
|  | CHEMBL614110 | LXFL 529 |
|  | CHEMBL614451 | SNB-75 |
|  | CHEMBL613977 | SF-268 |
|  | CHEMBL2366505 | Integrase |
|  | CHEMBL614643 | HOP-62 |
|  | CHEMBL1287610 | Neuraminidase |
|  | CHEMBL613533 | XF498 |
|  | CHEMBL5697 | Egl nine homolog 1 |
|  | CHEMBL3577 | Aldehyde dehydrogenase 1A1 |
|  | CHEMBL1293231 | Nuclear receptor ROR-gamma |
|  | CHEMBL614925 | SK-OV-3 |
|  | CHEMBL614791 | KM-20L2 |
|  | CHEMBL612263 | HCT-15 |
|  | CHEMBL5983 | Aldo-keto reductase family 1 member B10 |
|  | CHEMBL614740 | NCI-H226 |
|  | CHEMBL613739 | Vesicular stomatitis virus |
|  | CHEMBL375 | Mus musculus |
|  | CHEMBL614917 | SK-MEL-2 |
|  | CHEMBL2366755 | Human herpesvirus 2 strain G |
|  | CHEMBL221 | Cyclooxygenase-1 |
|  | CHEMBL614487 | EKVX |
|  | CHEMBL612927 | Mammalian orthoreovirus 1 |
|  | CHEMBL614056 | TK-10 |
|  | CHEMBL614908 | SF-295 |
|  | CHEMBL2524 | Alpha-galactosidase A |
| ferulic acid | CHEMBL2367188 | Eutypa lata |
|  | CHEMBL2366839 | Neofusicoccum luteum |
|  | CHEMBL612715 | Pisum sativum |
|  | CHEMBL4789 | Carbonic anhydrase VA |
|  | CHEMBL612788 | Lasiodiplodia theobromae |
|  | CHEMBL261 | Carbonic anhydrase I |
|  | CHEMBL4358 | Arachidonate 15-lipoxygenase |
|  | CHEMBL2367030 | Botryosphaeria dothidea |
|  | CHEMBL2367242 | Neofusicoccum ribis |
|  | CHEMBL2146309 | ATP-dependent Clp protease proteolytic subunit |
|  | CHEMBL3318 | Tyrosinase |
|  | CHEMBL2366838 | Diplodia mutila |
|  | CHEMBL4078 | Acetylcholinesterase |
|  | CHEMBL2367357 | Leaf |
|  | CHEMBL5077 | Butyrylcholinesterase |
|  | CHEMBL2885 | Carbonic anhydrase III |
|  | CHEMBL2186 | Carbonic anhydrase XIII |
|  | CHEMBL612602 | Ralstonia solanacearum |
|  | CHEMBL2367189 | Togninia minima |
|  | CHEMBL3729 | Carbonic anhydrase IV |
|  | CHEMBL4357 | Alpha-glucosidase MAL62 |
|  | CHEMBL4528 | Hyaluronidase-1 |
|  | CHEMBL614051 | OVCAR-4 |
|  | CHEMBL2367106 | Neofusicoccum parvum |
|  | CHEMBL4445 | Serine/threonine protein phosphatase 2B catalytic subunit |
|  | CHEMBL5763 | Cholinesterase |
|  | CHEMBL2487 | Beta amyloid A4 protein |
|  | CHEMBL4801 | Caspase-1 |
|  | CHEMBL233 | Mu opioid receptor |
|  | CHEMBL614317 | M14 |
|  | CHEMBL1867 | Alpha-2a adrenergic receptor |
|  | CHEMBL1075138 | Tyrosyl-DNA phosphodiesterase 1 |
|  | CHEMBL5017 | Serotonin 4 (5-HT4) receptor |
|  | CHEMBL614919 | SK-MEL-28 |
|  | CHEMBL274 | C-C chemokine receptor type 5 |
|  | CHEMBL1951 | Monoamine oxidase A |
|  | CHEMBL392 | A549 |
|  | CHEMBL366 | Candida albicans |
|  | CHEMBL4074 | Angiotensin-converting enzyme |
|  | CHEMBL3385 | MAP kinase ERK1 |
|  | CHEMBL4071 | Cathepsin G |
|  | CHEMBL3619 | UDP-glucuronosyltransferase 1A4 |
|  | CHEMBL614067 | CAKI-1 |
|  | CHEMBL613508 | DU-145 |
|  | CHEMBL384 | HT-29 |
|  | CHEMBL1287617 | UDP-glucuronosyltransferase 1-1 |
|  | CHEMBL4777 | Neuropeptide Y receptor type 1 |
|  | CHEMBL1889 | Vasopressin V1a receptor |
|  | CHEMBL378 | Human immunodeficiency virus 1 |
|  | CHEMBL613102 | 786-0 |
|  | CHEMBL245 | Muscarinic acetylcholine receptor M3 |
|  | CHEMBL1832 | Calcitonin receptor |
|  | CHEMBL614803 | NCI-H322M |
|  | CHEMBL614793 | L6 |
|  | CHEMBL1901 | Cholecystokinin A receptor |
|  | CHEMBL614316 | HT-22 |
|  | CHEMBL3879801 | NON-PROTEIN TARGET |
|  | CHEMBL4607 | Angiotensin II type 2 (AT-2) receptor |
|  | CHEMBL382 | CCRF-CEM |
|  | CHEMBL2367441 | Root |
|  | CHEMBL231 | Histamine H1 receptor |
|  | CHEMBL315 | Alpha-1b adrenergic receptor |
|  | CHEMBL390 | PC-3 |
|  | CHEMBL4018 | Neuropeptide Y receptor type 2 |
|  | CHEMBL287 | Sigma opioid receptor |
|  | CHEMBL1827 | Phosphodiesterase 5A |
|  | CHEMBL203 | Epidermal growth factor receptor erbB1 |
|  | CHEMBL613453 | Meloidogyne incognita |
|  | CHEMBL204 | Thrombin |
|  | CHEMBL352 | Staphylococcus aureus |
|  | CHEMBL612555 | OVCAR-8 |
|  | CHEMBL613900 | Athelia rolfsii |
|  | CHEMBL2367291 | Phaeomoniella chlamydospora |
|  | CHEMBL222 | Norepinephrine transporter |
|  | CHEMBL614164 | SNB-19 |
|  | CHEMBL614214 | OVCAR-5 |
|  | CHEMBL217 | Dopamine D2 receptor |
|  | CHEMBL3969 | Carbonic anhydrase VB |
|  | CHEMBL237 | Kappa opioid receptor |
|  | CHEMBL3048 | Nitric-oxide synthase |
|  | CHEMBL1293226 | Lysine-specific demethylase 4D-like |
|  | CHEMBL614860 | SF-539 |
|  | CHEMBL400 | MDA-MB-231 |
|  | CHEMBL613984 | IGROV-1 |
|  | CHEMBL2434 | Interleukin-8 receptor B |
|  | CHEMBL1798 | Cysteinyl leukotriene receptor 1 |
|  | CHEMBL391 | Vero |
|  | CHEMBL230 | Cyclooxygenase-2 |
|  | CHEMBL4029 | Interleukin-8 receptor A |
|  | CHEMBL613855 | HCC 2998 |
|  | CHEMBL289 | Cytochrome P450 2D6 |
|  | CHEMBL3392921 | Glycine receptor |
|  | CHEMBL612870 | Candida tropicalis |
|  | CHEMBL3025 | Carbonic anhydrase VI |
|  | CHEMBL1942 | Alpha-2b adrenergic receptor |
|  | CHEMBL612796 | UACC-257 |
|  | CHEMBL614647 | WiDr |
|  | CHEMBL614096 | LOX IMVI |
|  | CHEMBL228 | Serotonin transporter |
|  | CHEMBL3559673 | 2-heptyl-4(1H)-quinolone synthase PqsD |
|  | CHEMBL246 | Beta-3 adrenergic receptor |
|  | CHEMBL1973 | Tyrosinase |
|  | CHEMBL3459 | Serotonin 1b (5-HT1b) receptor |
|  | CHEMBL2327 | Neurokinin 2 receptor |
|  | CHEMBL388 | MT4 |
|  | CHEMBL252 | Endothelin receptor ET-A |
|  | CHEMBL5282 | Cytochrome P450 2A6 |
|  | CHEMBL2414 | C-C chemokine receptor type 4 |
|  | CHEMBL3155 | Serotonin 7 (5-HT7) receptor |
|  | CHEMBL394 | HCT-116 |
|  | CHEMBL2366283 | C2BBe1 |
|  | CHEMBL340 | Cytochrome P450 3A4 |
|  | CHEMBL256 | Adenosine A3 receptor |
|  | CHEMBL1914 | Butyrylcholinesterase |
|  | CHEMBL319 | Alpha-1a adrenergic receptor |
|  | CHEMBL3371 | Serotonin 6 (5-HT6) receptor |
|  | CHEMBL3622 | Cytochrome P450 2C19 |
|  | CHEMBL614882 | RPMI-8226 |
|  | CHEMBL613606 | Aorta |
|  | CHEMBL1868 | Vascular endothelial growth factor receptor 1 |
|  | CHEMBL321 | Matrix metalloproteinase 9 |
|  | CHEMBL259 | Melanocortin receptor 4 |
|  | CHEMBL2367281 | Pythium |
|  | CHEMBL614387 | NCI-H522 |
|  | CHEMBL2034 | Glucocorticoid receptor |
|  | CHEMBL387 | MCF7 |
|  | CHEMBL211 | Muscarinic acetylcholine receptor M2 |
|  | CHEMBL3356 | Cytochrome P450 1A2 |
|  | CHEMBL236 | Delta opioid receptor |
|  | CHEMBL614697 | MDA-MB-435 |
|  | CHEMBL213 | Beta-1 adrenergic receptor |
|  | CHEMBL4040 | MAP kinase ERK2 |
|  | CHEMBL613889 | HOP-92 |
|  | CHEMBL258 | Tyrosine-protein kinase LCK |
|  | CHEMBL4608 | Melanocortin receptor 5 |
|  | CHEMBL1929 | Xanthine dehydrogenase |
|  | CHEMBL2056 | Dopamine D1 receptor |
|  | CHEMBL615022 | U-251 |
|  | CHEMBL240 | HERG |
|  | CHEMBL3510 | Carbonic anhydrase XIV |
|  | CHEMBL3199 | Acetylcholinesterase |
|  | CHEMBL614213 | OVCAR-3 |
|  | CHEMBL612545 | Unchecked |
|  | CHEMBL1821 | Muscarinic acetylcholine receptor M4 |
|  | CHEMBL396 | NCI-H460 |
|  | CHEMBL242 | Estrogen receptor beta |
|  | CHEMBL614021 | Malme-3M |
|  | CHEMBL614177 | MOLT-4 |
|  | CHEMBL614561 | COLO 205 |
|  | CHEMBL614054 | SN12C |
|  | CHEMBL216 | Muscarinic acetylcholine receptor M1 |
|  | CHEMBL2326 | Carbonic anhydrase VII |
|  | CHEMBL3464 | Nitric oxide synthase |
|  | CHEMBL249 | Neurokinin 1 receptor |
|  | CHEMBL614645 | Hs-578T |
|  | CHEMBL6161 | UDP-glucuronosyltransferase 2B15 |
|  | CHEMBL614922 | SK-MEL-5 |
|  | CHEMBL238 | Dopamine transporter |
|  | CHEMBL2362975 | No relevant target |
|  | CHEMBL612262 | SW-620 |
|  | CHEMBL3594 | Carbonic anhydrase IX |
|  | CHEMBL3072 | Androgen Receptor |
|  | CHEMBL206 | Estrogen receptor alpha |
|  | CHEMBL332 | Matrix metalloproteinase-1 |
|  | CHEMBL5144 | Vasoactive intestinal polypeptide receptor 1 |
|  | CHEMBL613712 | Radical scavenging activity |
|  | CHEMBL3242 | Carbonic anhydrase XII |
|  | CHEMBL260 | MAP kinase p38 alpha |
|  | CHEMBL614721 | LoVo |
|  | CHEMBL299 | Protein kinase C alpha |
|  | CHEMBL1293278 | Geminin |
|  | CHEMBL2366750 | Diplodia seriata |
|  | CHEMBL224 | Serotonin 2a (5-HT2a) receptor |
|  | CHEMBL1824 | Receptor protein-tyrosine kinase erbB-2 |
|  | CHEMBL3514 | LDL-associated phospholipase A2 |
|  | CHEMBL376 | Rattus norvegicus |
|  | CHEMBL2093865 | Histone deacetylase |
|  | CHEMBL1697668 | Solute carrier organic anion transporter family member 1B1 |
|  | CHEMBL402 | HMG-CoA reductase |
|  | CHEMBL354 | Escherichia coli |
|  | CHEMBL612558 | ADMET |
|  | CHEMBL219 | Dopamine D4 receptor |
|  | CHEMBL614709 | KM12 |
|  | CHEMBL223 | Alpha-1d adrenergic receptor |
|  | CHEMBL1833 | Serotonin 2b (5-HT2b) receptor |
|  | CHEMBL614388 | UO-31 |
|  | CHEMBL614361 | T47D |
|  | CHEMBL614997 | NCI-H23 |
|  | CHEMBL383 | HL-60 |
|  | CHEMBL3157 | Bradykinin B2 receptor |
|  | CHEMBL1941 | Histamine H2 receptor |
|  | CHEMBL2622 | Aldose reductase |
|  | CHEMBL3397 | Cytochrome P450 2C9 |
|  | CHEMBL218 | Cannabinoid CB1 receptor |
|  | CHEMBL1793 | Parathyroid hormone receptor |
|  | CHEMBL3785 | Hydroxycarboxylic acid receptor 2 |
|  | CHEMBL251 | Adenosine A2a receptor |
|  | CHEMBL614078 | MDA-N |
|  | CHEMBL614139 | PANC-1 |
|  | CHEMBL5281 | Cytochrome P450 2E1 |
|  | CHEMBL4332 | Quinone oxidoreductase |
|  | CHEMBL1835 | Thromboxane-A synthase |
|  | CHEMBL612647 | Candida glabrata |
|  | CHEMBL1743121 | Solute carrier organic anion transporter family member 1B3 |
|  | CHEMBL205 | Carbonic anhydrase II |
|  | CHEMBL614300 | SR |
|  | CHEMBL248 | Leukocyte elastase |
|  | CHEMBL613829 | NCI/ADR-RES |
|  | CHEMBL356 | Streptococcus pyogenes |
|  | CHEMBL234 | Dopamine D3 receptor |
|  | CHEMBL250 | Platelet activating factor receptor |
|  | CHEMBL1841 | Tyrosine-protein kinase FYN |
|  | CHEMBL1916 | Alpha-2c adrenergic receptor |
|  | CHEMBL614519 | ACHN |
|  | CHEMBL385 | K562 |
|  | CHEMBL2035 | Muscarinic acetylcholine receptor M5 |
|  | CHEMBL612557 | RAW264.7 |
|  | CHEMBL4015 | C-C chemokine receptor type 2 |
|  | CHEMBL220 | Acetylcholinesterase |
|  | CHEMBL614451 | SNB-75 |
|  | CHEMBL613977 | SF-268 |
|  | CHEMBL225 | Serotonin 2c (5-HT2c) receptor |
|  | CHEMBL2366505 | Integrase |
|  | CHEMBL614643 | HOP-62 |
|  | CHEMBL4644 | Melanocortin receptor 3 |
|  | CHEMBL5486 | Insulin receptor |
|  | CHEMBL612263 | HCT-15 |
|  | CHEMBL614740 | NCI-H226 |
|  | CHEMBL375 | Mus musculus |
|  | CHEMBL614917 | SK-MEL-2 |
|  | CHEMBL226 | Adenosine A1 receptor |
|  | CHEMBL1909043 | Leukotriene C4 synthase |
|  | CHEMBL210 | Beta-2 adrenergic receptor |
|  | CHEMBL221 | Cyclooxygenase-1 |
|  | CHEMBL3243 | Leukocyte common antigen |
|  | CHEMBL1909044 | Progesterone receptor |
|  | CHEMBL614487 | EKVX |
|  | CHEMBL273 | Serotonin 1a (5-HT1a) receptor |
|  | CHEMBL614056 | TK-10 |
|  | CHEMBL614908 | SF-295 |
| Isoferulic acid | CHEMBL4377 | Guanine nucleotide-binding protein G(s) |
|  | CHEMBL1741209 | ATPase family AAA domain-containing protein 5 |
|  | CHEMBL3318 | Tyrosinase |
|  | CHEMBL612602 | Ralstonia solanacearum |
|  | CHEMBL4528 | Hyaluronidase-1 |
|  | CHEMBL1075138 | Tyrosyl-DNA phosphodiesterase 1 |
|  | CHEMBL399 | HeLa |
|  | CHEMBL392 | A549 |
|  | CHEMBL3879801 | NON-PROTEIN TARGET |
|  | CHEMBL1741193 | Chromobox protein homolog 1 |
|  | CHEMBL612545 | Unchecked |
|  | CHEMBL2362975 | No relevant target |
|  | CHEMBL206 | Estrogen receptor alpha |
|  | CHEMBL613712 | Radical scavenging activity |
|  | CHEMBL612557 | RAW264.7 |
|  | CHEMBL614580 | HT-1080 |
| Rosmarinic acid | CHEMBL1075175 | 72 kDa type IV collagenase |
|  | CHEMBL1741209 | ATPase family AAA domain-containing protein 5 |
|  | CHEMBL1293236 | ATP-dependent DNA helicase Q1 |
|  | CHEMBL1293237 | Bloom syndrome protein |
|  | CHEMBL1293303 | Nonstructural protein 1 |
|  | CHEMBL3318 | Tyrosinase |
|  | CHEMBL4078 | Acetylcholinesterase |
|  | CHEMBL2392 | DNA polymerase beta |
|  | CHEMBL1293234 | Putative fructose-1 |
|  | CHEMBL613907 | Bacillus subtilis subsp. spizizenii |
|  | CHEMBL6110 | Thioredoxin glutathione reductase |
|  | CHEMBL1293297 | Bifunctional protein glmU |
|  | CHEMBL5686 | 6-phospho-1-fructokinase |
|  | CHEMBL4528 | Hyaluronidase-1 |
|  | CHEMBL5763 | Cholinesterase |
|  | CHEMBL2487 | Beta amyloid A4 protein |
|  | CHEMBL1075138 | Tyrosyl-DNA phosphodiesterase 1 |
|  | CHEMBL615021 | U251 |
|  | CHEMBL612679 | Respiratory syncytial virus |
|  | CHEMBL366 | Candida albicans |
|  | CHEMBL2366516 | Reverse transcriptase |
|  | CHEMBL5817 | Signal transducer and activator of transcription 5B |
|  | CHEMBL378 | Human immunodeficiency virus 1 |
|  | CHEMBL1075189 | Pyruvate kinase isozymes M1/M2 |
|  | CHEMBL3879801 | NON-PROTEIN TARGET |
|  | CHEMBL397 | Jurkat |
|  | CHEMBL1293227 | Ubiquitin carboxyl-terminal hydrolase 2 |
|  | CHEMBL352 | Staphylococcus aureus |
|  | CHEMBL1947 | Thyroid hormone receptor beta-1 |
|  | CHEMBL1900 | Aldose reductase |
|  | CHEMBL2026 | Beta-lactamase AmpC |
|  | CHEMBL1293226 | Lysine-specific demethylase 4D-like |
|  | CHEMBL348 | Pseudomonas aeruginosa |
|  | CHEMBL230 | Cyclooxygenase-2 |
|  | CHEMBL3471 | Human immunodeficiency virus type 1 integrase |
|  | CHEMBL2179 | Beta-glucocerebrosidase |
|  | CHEMBL1741220 | Bromodomain adjacent to zinc finger domain protein 2B |
|  | CHEMBL395 | HepG2 |
|  | CHEMBL388 | MT4 |
|  | CHEMBL612546 | Molecular identity unknown |
|  | CHEMBL1293248 | 4'-phosphopantetheinyl transferase ffp |
|  | CHEMBL4977 | Proto-oncogene c-JUN |
|  | CHEMBL258 | Tyrosine-protein kinase LCK |
|  | CHEMBL353 | Staphylococcus epidermidis |
|  | CHEMBL614781 | BV-2 |
|  | CHEMBL4026 | Signal transducer and activator of transcription 3 |
|  | CHEMBL614657 | C6 |
|  | CHEMBL612545 | Unchecked |
|  | CHEMBL3194 | Transthyretin |
|  | CHEMBL2362975 | No relevant target |
|  | CHEMBL614818 | HEK293 |
|  | CHEMBL332 | Matrix metalloproteinase-1 |
|  | CHEMBL613712 | Radical scavenging activity |
|  | CHEMBL2608 | Lysosomal alpha-glucosidase |
|  | CHEMBL3514 | LDL-associated phospholipase A2 |
|  | CHEMBL376 | Rattus norvegicus |
|  | CHEMBL1697668 | Solute carrier organic anion transporter family member 1B1 |
|  | CHEMBL613729 | Japanese encephalitis virus |
|  | CHEMBL5619 | DNA-(apurinic or apyrimidinic site) lyase |
|  | CHEMBL354 | Escherichia coli |
|  | CHEMBL612558 | ADMET |
|  | CHEMBL358 | Aspergillus niger |
|  | CHEMBL6101 | Signal transducer and activator of transcription 1-alpha/beta |
|  | CHEMBL2622 | Aldose reductase |
|  | CHEMBL1293224 | Microtubule-associated protein tau |
|  | CHEMBL1743121 | Solute carrier organic anion transporter family member 1B3 |
|  | CHEMBL364 | Plasmodium falciparum |
|  | CHEMBL1841 | Tyrosine-protein kinase FYN |
|  | CHEMBL2366505 | Integrase |
|  | CHEMBL4361 | Induced myeloid leukemia cell differentiation protein Mcl-1 |
|  | CHEMBL375 | Mus musculus |
|  | CHEMBL221 | Cyclooxygenase-1 |
|  | CHEMBL267 | Tyrosine-protein kinase SRC |
| Vanillic Acid | CHEMBL2366839 | Neofusicoccum luteum |
|  | CHEMBL612788 | Lasiodiplodia theobromae |
|  | CHEMBL2367030 | Botryosphaeria dothidea |
|  | CHEMBL4150 | Enoyl-acyl-carrier protein reductase |
|  | CHEMBL2367001 | Hylobius abietis |
|  | CHEMBL2367242 | Neofusicoccum ribis |
|  | CHEMBL5391 | DNA polymerase iota |
|  | CHEMBL3318 | Tyrosinase |
|  | CHEMBL2366838 | Diplodia mutila |
|  | CHEMBL1687677 | Pancreatic triacylglycerol lipase |
|  | CHEMBL2367189 | Togninia minima |
|  | CHEMBL2367106 | Neofusicoccum parvum |
|  | CHEMBL614919 | SK-MEL-28 |
|  | CHEMBL392 | A549 |
|  | CHEMBL613979 | HUVEC |
|  | CHEMBL384 | HT-29 |
|  | CHEMBL2500 | Thiopurine S-methyltransferase |
|  | CHEMBL3879801 | NON-PROTEIN TARGET |
|  | CHEMBL390 | PC-3 |
|  | CHEMBL1075556 | OE33 |
|  | CHEMBL613453 | Meloidogyne incognita |
|  | CHEMBL614775 | T98G |
|  | CHEMBL352 | Staphylococcus aureus |
|  | CHEMBL2366427 | Polyphenol oxidase |
|  | CHEMBL1900 | Aldose reductase |
|  | CHEMBL2026 | Beta-lactamase AmpC |
|  | CHEMBL387 | MCF7 |
|  | CHEMBL612656 | B16-F10 |
|  | CHEMBL612545 | Unchecked |
|  | CHEMBL360 | Mycobacterium tuberculosis |
|  | CHEMBL2362975 | No relevant target |
|  | CHEMBL206 | Estrogen receptor alpha |
|  | CHEMBL613712 | Radical scavenging activity |
|  | CHEMBL5514 | Huntingtin |
|  | CHEMBL614721 | LoVo |
|  | CHEMBL614632 | RBL-2H3 |
|  | CHEMBL2366750 | Diplodia seriata |
|  | CHEMBL2093865 | Histone deacetylase |
|  | CHEMBL612558 | ADMET |
|  | CHEMBL612557 | RAW264.7 |
|  | CHEMBL614628 | Raji |
|  | CHEMBL613533 | XF498 |
|  | CHEMBL614925 | SK-OV-3 |
|  | CHEMBL375 | Mus musculus |
|  | CHEMBL614917 | SK-MEL-2 |
| Protocatechuic acid | CHEMBL1293263 | M18 aspartyl aminopeptidase |
|  | CHEMBL261 | Carbonic anhydrase I |
|  | CHEMBL2093862 | Runt-related transcription factor 1/Core-binding factor subunit beta |
|  | CHEMBL5391 | DNA polymerase iota |
|  | CHEMBL3318 | Tyrosinase |
|  | CHEMBL2392 | DNA polymerase beta |
|  | CHEMBL3554 | 3-dehydroquinate synthase |
|  | CHEMBL3729 | Carbonic anhydrase IV |
|  | CHEMBL613564 | Brain |
|  | CHEMBL2366635 | Heterodera zeae |
|  | CHEMBL613140 | Brugia malayi |
|  | CHEMBL215 | Arachidonate 5-lipoxygenase |
|  | CHEMBL392 | A549 |
|  | CHEMBL366 | Candida albicans |
|  | CHEMBL4588 | Matrix metalloproteinase 8 |
|  | CHEMBL2146302 | Glutaminase kidney isoform |
|  | CHEMBL3837 | Cathepsin L |
|  | CHEMBL613864 | Gibberella zeae |
|  | CHEMBL378 | Human immunodeficiency virus 1 |
|  | CHEMBL3890 | Selectin E |
|  | CHEMBL1287622 | Lethal(3)malignant brain tumor-like protein 1 |
|  | CHEMBL3879801 | NON-PROTEIN TARGET |
|  | CHEMBL613831 | SMMC-7721 |
|  | CHEMBL613453 | Meloidogyne incognita |
|  | CHEMBL352 | Staphylococcus aureus |
|  | CHEMBL2366427 | Polyphenol oxidase |
|  | CHEMBL1978 | Cytochrome P450 19A1 |
|  | CHEMBL613580 | Liver |
|  | CHEMBL1900 | Aldose reductase |
|  | CHEMBL1293226 | Lysine-specific demethylase 4D-like |
|  | CHEMBL4093 | LXR-beta |
|  | CHEMBL348 | Pseudomonas aeruginosa |
|  | CHEMBL283 | Matrix metalloproteinase 3 |
|  | CHEMBL614637 | KETR3 |
|  | CHEMBL333 | Matrix metalloproteinase-2 |
|  | CHEMBL3025 | Carbonic anhydrase VI |
|  | CHEMBL612544 | SW480 |
|  | CHEMBL1795125 | Carbonic anhydrase |
|  | CHEMBL614955 | Aspergillus flavus |
|  | CHEMBL321 | Matrix metalloproteinase 9 |
|  | CHEMBL1293248 | 4'-phosphopantetheinyl transferase ffp |
|  | CHEMBL387 | MCF7 |
|  | CHEMBL2808 | LXR-alpha |
|  | CHEMBL3253 | Serum albumin |
|  | CHEMBL3510 | Carbonic anhydrase XIV |
|  | CHEMBL361 | Saccharomyces cerevisiae |
|  | CHEMBL612545 | Unchecked |
|  | CHEMBL359 | Bacillus subtilis |
|  | CHEMBL2029197 | Rap guanine nucleotide exchange factor 3 |
|  | CHEMBL5346 | Tyrosinase |
|  | CHEMBL2326 | Carbonic anhydrase VII |
|  | CHEMBL360 | Mycobacterium tuberculosis |
|  | CHEMBL2362975 | No relevant target |
|  | CHEMBL614578 | Hs68 |
|  | CHEMBL3594 | Carbonic anhydrase IX |
|  | CHEMBL206 | Estrogen receptor alpha |
|  | CHEMBL332 | Matrix metalloproteinase-1 |
|  | CHEMBL613712 | Radical scavenging activity |
|  | CHEMBL3629 | Casein kinase II alpha |
|  | CHEMBL3242 | Carbonic anhydrase XII |
|  | CHEMBL1293278 | Geminin |
|  | CHEMBL2608 | Lysosomal alpha-glucosidase |
|  | CHEMBL3161 | Leukocyte adhesion molecule-1 |
|  | CHEMBL3514 | LDL-associated phospholipase A2 |
|  | CHEMBL354 | Escherichia coli |
|  | CHEMBL612558 | ADMET |
|  | CHEMBL381 | B16 |
|  | CHEMBL358 | Aspergillus niger |
|  | CHEMBL383 | HL-60 |
|  | CHEMBL1293224 | Microtubule-associated protein tau |
|  | CHEMBL4372 | Anthrax lethal factor |
|  | CHEMBL205 | Carbonic anhydrase II |
|  | CHEMBL364 | Plasmodium falciparum |
|  | CHEMBL4662 | Proteasome Macropain subunit MB1 |
|  | CHEMBL5378 | P-selectin |
|  | CHEMBL2366505 | Integrase |
|  | CHEMBL5697 | Egl nine homolog 1 |
| Gallic acid | CHEMBL1293235 | Prelamin-A/C |
|  | CHEMBL5365 | DNA polymerase kappa |
|  | CHEMBL614614 | Anabaena flos-aquae |
|  | CHEMBL2366839 | Neofusicoccum luteum |
|  | CHEMBL613162 | Trichophyton mentagrophytes |
|  | CHEMBL612649 | Trichophyton rubrum |
|  | CHEMBL6100 | Autoinducer 2-binding periplasmic protein luxP |
|  | CHEMBL4996 | Fucosyltransferase 4 |
|  | CHEMBL4789 | Carbonic anhydrase VA |
|  | CHEMBL612788 | Lasiodiplodia theobromae |
|  | CHEMBL261 | Carbonic anhydrase I |
|  | CHEMBL612744 | Feline coronavirus |
|  | CHEMBL4159 | Endoplasmic reticulum-associated amyloid beta-peptide-binding protein |
|  | CHEMBL2367030 | Botryosphaeria dothidea |
|  | CHEMBL2146316 | Putative uncharacterized protein |
|  | CHEMBL3232678 | Neuraminidase |
|  | CHEMBL3318 | Tyrosinase |
|  | CHEMBL613174 | Vibrio harveyi |
|  | CHEMBL2366838 | Diplodia mutila |
|  | CHEMBL1293234 | Putative fructose-1 |
|  | CHEMBL612341 | Artemia salina |
|  | CHEMBL1687677 | Pancreatic triacylglycerol lipase |
|  | CHEMBL614414 | Porphyromonas gingivalis |
|  | CHEMBL2885 | Carbonic anhydrase III |
|  | CHEMBL613078 | Actinomyces viscosus |
|  | CHEMBL2186 | Carbonic anhydrase XIII |
|  | CHEMBL612602 | Ralstonia solanacearum |
|  | CHEMBL612942 | Microcystis aeruginosa |
|  | CHEMBL2367189 | Togninia minima |
|  | CHEMBL612990 | Pectobacterium carotovorum |
|  | CHEMBL3729 | Carbonic anhydrase IV |
|  | CHEMBL4357 | Alpha-glucosidase MAL62 |
|  | CHEMBL5686 | 6-phospho-1-fructokinase |
|  | CHEMBL2366635 | Heterodera zeae |
|  | CHEMBL2367106 | Neofusicoccum parvum |
|  | CHEMBL2487 | Beta amyloid A4 protein |
|  | CHEMBL393 | CEM |
|  | CHEMBL1075138 | Tyrosyl-DNA phosphodiesterase 1 |
|  | CHEMBL4241 | Squalene monooxygenase |
|  | CHEMBL399 | HeLa |
|  | CHEMBL612679 | Respiratory syncytial virus |
|  | CHEMBL392 | A549 |
|  | CHEMBL366 | Candida albicans |
|  | CHEMBL363 | Aspergillus fumigatus |
|  | CHEMBL3717 | Hepatocyte growth factor receptor |
|  | CHEMBL377 | Human herpesvirus 1 |
|  | CHEMBL2146302 | Glutaminase kidney isoform |
|  | CHEMBL613903 | Babesia gibsoni |
|  | CHEMBL2367089 | Unidentified Influenza A virus (H1N2) |
|  | CHEMBL3286066 | Solute carrier family 2 |
|  | CHEMBL386 | L1210 |
|  | CHEMBL1287617 | UDP-glucuronosyltransferase 1-1 |
|  | CHEMBL247 | Human immunodeficiency virus type 1 reverse transcriptase |
|  | CHEMBL378 | Human immunodeficiency virus 1 |
|  | CHEMBL3890 | Selectin E |
|  | CHEMBL613863 | Bacillus sphaericus |
|  | CHEMBL1977 | Vitamin D receptor |
|  | CHEMBL3879801 | NON-PROTEIN TARGET |
|  | CHEMBL382 | CCRF-CEM |
|  | CHEMBL612945 | Microsporum gypseum |
|  | CHEMBL612386 | Epidermophyton floccosum |
|  | CHEMBL613453 | Meloidogyne incognita |
|  | CHEMBL204 | Thrombin |
|  | CHEMBL613126 | Human parainfluenza virus 3 |
|  | CHEMBL352 | Staphylococcus aureus |
|  | CHEMBL3596077 | Alpha-(1 |
|  | CHEMBL1978 | Cytochrome P450 19A1 |
|  | CHEMBL2366902 | Influenza A virus H3N2 |
|  | CHEMBL1900 | Aldose reductase |
|  | CHEMBL3969 | Carbonic anhydrase VB |
|  | CHEMBL1293226 | Lysine-specific demethylase 4D-like |
|  | CHEMBL400 | MDA-MB-231 |
|  | CHEMBL2157 | Interleukin-8 |
|  | CHEMBL391 | Vero |
|  | CHEMBL348 | Pseudomonas aeruginosa |
|  | CHEMBL612743 | Felid herpesvirus 1 |
|  | CHEMBL230 | Cyclooxygenase-2 |
|  | CHEMBL6032 | Histone-lysine N-methyltransferase |
|  | CHEMBL612870 | Candida tropicalis |
|  | CHEMBL3025 | Carbonic anhydrase VI |
|  | CHEMBL612320 | Human coxsackievirus B4 |
|  | CHEMBL615029 | Corynebacterium accolens |
|  | CHEMBL1795125 | Carbonic anhydrase |
|  | CHEMBL389 | P388 |
|  | CHEMBL612546 | Molecular identity unknown |
|  | CHEMBL614955 | Aspergillus flavus |
|  | CHEMBL2366865 | Human immunodeficiency virus type 2 (ISOLATE ROD) |
|  | CHEMBL613606 | Aorta |
|  | CHEMBL613853 | CHO |
|  | CHEMBL5501 | Histone acetyltransferase GCN5 |
|  | CHEMBL2034 | Glucocorticoid receptor |
|  | CHEMBL1293248 | 4'-phosphopantetheinyl transferase ffp |
|  | CHEMBL613135 | Caenorhabditis elegans |
|  | CHEMBL4040 | MAP kinase ERK2 |
|  | CHEMBL4096 | Cellular tumor antigen p53 |
|  | CHEMBL4695 | Fructose-bisphosphate aldolase A |
|  | CHEMBL615022 | U-251 |
|  | CHEMBL3510 | Carbonic anhydrase XIV |
|  | CHEMBL361 | Saccharomyces cerevisiae |
|  | CHEMBL612513 | Heliothis virescens |
|  | CHEMBL612545 | Unchecked |
|  | CHEMBL359 | Bacillus subtilis |
|  | CHEMBL613741 | Punta Toro virus |
|  | CHEMBL614526 | BGC-823 |
|  | CHEMBL2326 | Carbonic anhydrase VII |
|  | CHEMBL3464 | Nitric oxide synthase |
|  | CHEMBL3706 | ADAM17 |
|  | CHEMBL2362975 | No relevant target |
|  | CHEMBL1075094 | Nuclear factor erythroid 2-related factor 2 |
|  | CHEMBL3594 | Carbonic anhydrase IX |
|  | CHEMBL206 | Estrogen receptor alpha |
|  | CHEMBL613712 | Radical scavenging activity |
|  | CHEMBL3242 | Carbonic anhydrase XII |
|  | CHEMBL1808 | Angiotensin-converting enzyme |
|  | CHEMBL613261 | Prevotella intermedia |
|  | CHEMBL1075148 | Nuclear factor erythroid 2-related factor 2 |
|  | CHEMBL2366750 | Diplodia seriata |
|  | CHEMBL3161 | Leukocyte adhesion molecule-1 |
|  | CHEMBL3514 | LDL-associated phospholipase A2 |
|  | CHEMBL614696 | Lymphoblastoid cells |
|  | CHEMBL1697668 | Solute carrier organic anion transporter family member 1B1 |
|  | CHEMBL613493 | Vaccinia virus |
|  | CHEMBL5619 | DNA-(apurinic or apyrimidinic site) lyase |
|  | CHEMBL354 | Escherichia coli |
|  | CHEMBL612558 | ADMET |
|  | CHEMBL2284 | Glyceraldehyde-3-phosphate dehydrogenase liver |
|  | CHEMBL358 | Aspergillus niger |
|  | CHEMBL383 | HL-60 |
|  | CHEMBL1293224 | Microtubule-associated protein tau |
|  | CHEMBL4372 | Anthrax lethal factor |
|  | CHEMBL1743121 | Solute carrier organic anion transporter family member 1B3 |
|  | CHEMBL205 | Carbonic anhydrase II |
|  | CHEMBL364 | Plasmodium falciparum |
|  | CHEMBL613725 | Sindbis virus |
|  | CHEMBL614535 | Calu-6 |
|  | CHEMBL1293255 | 15-hydroxyprostaglandin dehydrogenase [NAD+] |
|  | CHEMBL385 | K562 |
|  | CHEMBL613721 | Reovirus sp. |
|  | CHEMBL612557 | RAW264.7 |
|  | CHEMBL614510 | 3T3-L1 |
|  | CHEMBL5378 | P-selectin |
|  | CHEMBL614176 | MOLT-3 |
|  | CHEMBL3577 | Aldehyde dehydrogenase 1A1 |
|  | CHEMBL398 | KB |
|  | CHEMBL1293231 | Nuclear receptor ROR-gamma |
|  | CHEMBL613739 | Vesicular stomatitis virus |
|  | CHEMBL375 | Mus musculus |
|  | CHEMBL2007625 | Isocitrate dehydrogenase [NADP] cytoplasmic |
|  | CHEMBL1293232 | Survival motor neuron protein |
|  | CHEMBL612426 | Streptococcus mutans |
|  | CHEMBL365 | Filobasidiella neoformans |
|  | CHEMBL243 | Human immunodeficiency virus type 1 protease |
|  | CHEMBL612267 | L929 |
| Diethyl phthalate | CHEMBL2362981 | TAR DNA-binding protein 43 |
|  | CHEMBL2288 | Peptidyl-prolyl cis-trans isomerase NIMA-interacting 1 |
|  | CHEMBL1871 | Androgen Receptor |
|  | CHEMBL613071 | Bacillus megaterium |
|  | CHEMBL613187 | Chlorella fusca |
|  | CHEMBL1741193 | Chromobox protein homolog 1 |
|  | CHEMBL612546 | Molecular identity unknown |
|  | CHEMBL2034 | Glucocorticoid receptor |
|  | CHEMBL612545 | Unchecked |
|  | CHEMBL1293258 | Mothers against decapentaplegic homolog 3 |
|  | CHEMBL2362975 | No relevant target |
|  | CHEMBL1075094 | Nuclear factor erythroid 2-related factor 2 |
|  | CHEMBL1963 | Thyroid stimulating hormone receptor |
|  | CHEMBL1293278 | Geminin |
|  | CHEMBL614696 | Lymphoblastoid cells |
|  | CHEMBL612558 | ADMET |
|  | CHEMBL2007625 | Isocitrate dehydrogenase [NADP] cytoplasmic |
| Ligustilide | CHEMBL1293235 | Prelamin-A/C |
|  | CHEMBL5365 | DNA polymerase kappa |
|  | CHEMBL614614 | Anabaena flos-aquae |
|  | CHEMBL2366839 | Neofusicoccum luteum |
|  | CHEMBL613162 | Trichophyton mentagrophytes |
|  | CHEMBL612649 | Trichophyton rubrum |
|  | CHEMBL6100 | Autoinducer 2-binding periplasmic protein luxP |
|  | CHEMBL4996 | Fucosyltransferase 4 |
|  | CHEMBL4789 | Carbonic anhydrase VA |
|  | CHEMBL612788 | Lasiodiplodia theobromae |
|  | CHEMBL261 | Carbonic anhydrase I |
|  | CHEMBL612744 | Feline coronavirus |
|  | CHEMBL4159 | Endoplasmic reticulum-associated amyloid beta-peptide-binding protein |
|  | CHEMBL2367030 | Botryosphaeria dothidea |
|  | CHEMBL2146316 | Putative uncharacterized protein |
|  | CHEMBL3232678 | Neuraminidase |
|  | CHEMBL3318 | Tyrosinase |
|  | CHEMBL613174 | Vibrio harveyi |
|  | CHEMBL2366838 | Diplodia mutila |
|  | CHEMBL1293234 | Putative fructose-1 |
|  | CHEMBL612341 | Artemia salina |
|  | CHEMBL1687677 | Pancreatic triacylglycerol lipase |
|  | CHEMBL614414 | Porphyromonas gingivalis |
|  | CHEMBL2885 | Carbonic anhydrase III |
|  | CHEMBL613078 | Actinomyces viscosus |
|  | CHEMBL2186 | Carbonic anhydrase XIII |
|  | CHEMBL612602 | Ralstonia solanacearum |
|  | CHEMBL612942 | Microcystis aeruginosa |
|  | CHEMBL2367189 | Togninia minima |
|  | CHEMBL612990 | Pectobacterium carotovorum |
|  | CHEMBL3729 | Carbonic anhydrase IV |
|  | CHEMBL4357 | Alpha-glucosidase MAL62 |
|  | CHEMBL5686 | 6-phospho-1-fructokinase |
|  | CHEMBL2366635 | Heterodera zeae |
|  | CHEMBL2367106 | Neofusicoccum parvum |
|  | CHEMBL2487 | Beta amyloid A4 protein |
|  | CHEMBL393 | CEM |
|  | CHEMBL1075138 | Tyrosyl-DNA phosphodiesterase 1 |
|  | CHEMBL4241 | Squalene monooxygenase |
|  | CHEMBL399 | HeLa |
|  | CHEMBL612679 | Respiratory syncytial virus |
|  | CHEMBL392 | A549 |
|  | CHEMBL366 | Candida albicans |
|  | CHEMBL363 | Aspergillus fumigatus |
|  | CHEMBL3717 | Hepatocyte growth factor receptor |
|  | CHEMBL377 | Human herpesvirus 1 |
|  | CHEMBL2146302 | Glutaminase kidney isoform |
|  | CHEMBL613903 | Babesia gibsoni |
|  | CHEMBL2367089 | Unidentified Influenza A virus (H1N2) |
|  | CHEMBL3286066 | Solute carrier family 2 |
|  | CHEMBL386 | L1210 |
|  | CHEMBL1287617 | UDP-glucuronosyltransferase 1-1 |
|  | CHEMBL247 | Human immunodeficiency virus type 1 reverse transcriptase |
|  | CHEMBL378 | Human immunodeficiency virus 1 |
|  | CHEMBL3890 | Selectin E |
|  | CHEMBL613863 | Bacillus sphaericus |
|  | CHEMBL1977 | Vitamin D receptor |
|  | CHEMBL3879801 | NON-PROTEIN TARGET |
|  | CHEMBL382 | CCRF-CEM |
|  | CHEMBL612945 | Microsporum gypseum |
|  | CHEMBL612386 | Epidermophyton floccosum |
|  | CHEMBL613453 | Meloidogyne incognita |
|  | CHEMBL204 | Thrombin |
|  | CHEMBL613126 | Human parainfluenza virus 3 |
|  | CHEMBL352 | Staphylococcus aureus |
|  | CHEMBL3596077 | Alpha-(1 |
|  | CHEMBL1978 | Cytochrome P450 19A1 |
|  | CHEMBL2366902 | Influenza A virus H3N2 |
|  | CHEMBL1900 | Aldose reductase |
|  | CHEMBL3969 | Carbonic anhydrase VB |
|  | CHEMBL1293226 | Lysine-specific demethylase 4D-like |
|  | CHEMBL400 | MDA-MB-231 |
|  | CHEMBL2157 | Interleukin-8 |
|  | CHEMBL391 | Vero |
|  | CHEMBL348 | Pseudomonas aeruginosa |
|  | CHEMBL612743 | Felid herpesvirus 1 |
|  | CHEMBL230 | Cyclooxygenase-2 |
|  | CHEMBL6032 | Histone-lysine N-methyltransferase |
|  | CHEMBL612870 | Candida tropicalis |
|  | CHEMBL3025 | Carbonic anhydrase VI |
|  | CHEMBL612320 | Human coxsackievirus B4 |
|  | CHEMBL615029 | Corynebacterium accolens |
|  | CHEMBL1795125 | Carbonic anhydrase |
|  | CHEMBL389 | P388 |
|  | CHEMBL612546 | Molecular identity unknown |
|  | CHEMBL614955 | Aspergillus flavus |
|  | CHEMBL2366865 | Human immunodeficiency virus type 2 (ISOLATE ROD) |
|  | CHEMBL613606 | Aorta |
|  | CHEMBL613853 | CHO |
|  | CHEMBL5501 | Histone acetyltransferase GCN5 |
|  | CHEMBL2034 | Glucocorticoid receptor |
|  | CHEMBL1293248 | 4'-phosphopantetheinyl transferase ffp |
|  | CHEMBL613135 | Caenorhabditis elegans |
|  | CHEMBL4040 | MAP kinase ERK2 |
|  | CHEMBL4096 | Cellular tumor antigen p53 |
|  | CHEMBL4695 | Fructose-bisphosphate aldolase A |
|  | CHEMBL615022 | U-251 |
|  | CHEMBL3510 | Carbonic anhydrase XIV |
|  | CHEMBL361 | Saccharomyces cerevisiae |
|  | CHEMBL612513 | Heliothis virescens |
|  | CHEMBL612545 | Unchecked |
|  | CHEMBL359 | Bacillus subtilis |
|  | CHEMBL613741 | Punta Toro virus |
|  | CHEMBL614526 | BGC-823 |
|  | CHEMBL2326 | Carbonic anhydrase VII |
|  | CHEMBL3464 | Nitric oxide synthase |
|  | CHEMBL3706 | ADAM17 |
|  | CHEMBL2362975 | No relevant target |
|  | CHEMBL1075094 | Nuclear factor erythroid 2-related factor 2 |
|  | CHEMBL3594 | Carbonic anhydrase IX |
|  | CHEMBL206 | Estrogen receptor alpha |
|  | CHEMBL613712 | Radical scavenging activity |
|  | CHEMBL3242 | Carbonic anhydrase XII |
|  | CHEMBL1808 | Angiotensin-converting enzyme |
|  | CHEMBL613261 | Prevotella intermedia |
|  | CHEMBL1075148 | Nuclear factor erythroid 2-related factor 2 |
|  | CHEMBL2366750 | Diplodia seriata |
|  | CHEMBL3161 | Leukocyte adhesion molecule-1 |
|  | CHEMBL3514 | LDL-associated phospholipase A2 |
|  | CHEMBL614696 | Lymphoblastoid cells |
|  | CHEMBL1697668 | Solute carrier organic anion transporter family member 1B1 |
|  | CHEMBL613493 | Vaccinia virus |
|  | CHEMBL5619 | DNA-(apurinic or apyrimidinic site) lyase |
|  | CHEMBL354 | Escherichia coli |
|  | CHEMBL612558 | ADMET |
|  | CHEMBL2284 | Glyceraldehyde-3-phosphate dehydrogenase liver |
|  | CHEMBL358 | Aspergillus niger |
|  | CHEMBL383 | HL-60 |
|  | CHEMBL1293224 | Microtubule-associated protein tau |
|  | CHEMBL4372 | Anthrax lethal factor |
|  | CHEMBL1743121 | Solute carrier organic anion transporter family member 1B3 |
|  | CHEMBL205 | Carbonic anhydrase II |
|  | CHEMBL364 | Plasmodium falciparum |
|  | CHEMBL613725 | Sindbis virus |
|  | CHEMBL614535 | Calu-6 |
|  | CHEMBL1293255 | 15-hydroxyprostaglandin dehydrogenase [NAD+] |
|  | CHEMBL385 | K562 |
|  | CHEMBL613721 | Reovirus sp. |
|  | CHEMBL612557 | RAW264.7 |
|  | CHEMBL614510 | 3T3-L1 |
|  | CHEMBL5378 | P-selectin |
|  | CHEMBL614176 | MOLT-3 |
|  | CHEMBL3577 | Aldehyde dehydrogenase 1A1 |
|  | CHEMBL398 | KB |
|  | CHEMBL1293231 | Nuclear receptor ROR-gamma |
|  | CHEMBL613739 | Vesicular stomatitis virus |
|  | CHEMBL375 | Mus musculus |
|  | CHEMBL2007625 | Isocitrate dehydrogenase [NADP] cytoplasmic |
|  | CHEMBL1293232 | Survival motor neuron protein |
|  | CHEMBL612426 | Streptococcus mutans |
|  | CHEMBL365 | Filobasidiella neoformans |
|  | CHEMBL243 | Human immunodeficiency virus type 1 protease |
|  | CHEMBL612267 | L929 |
